# SomaticCombiner: improving the performance of somatic variant calling based on evaluation tests and a consensus approach

**DOI:** 10.1038/s41598-020-69772-8

**Published:** 2020-07-30

**Authors:** Mingyi Wang, Wen Luo, Kristine Jones, Xiaopeng Bian, Russell Williams, Herbert Higson, Dongjing Wu, Belynda Hicks, Meredith Yeager, Bin Zhu

**Affiliations:** 1 Cancer Genomics Research Laboratory, Division of Cancer Epidemiology and Genetics, Frederick National Laboratory for Cancer Research, Frederick, MD 20877 USA; 2 Center for Biomedical Informatics and Information Technology, National Cancer Institute, Rockville, MD 20850 USA

**Keywords:** Cancer, Computational biology and bioinformatics

## Abstract

It is challenging to identify somatic variants from high-throughput sequence reads due to tumor heterogeneity, sub-clonality, and sequencing artifacts. In this study, we evaluated the performance of eight primary somatic variant callers and multiple ensemble methods using both real and synthetic whole-genome sequencing, whole-exome sequencing, and deep targeted sequencing datasets with the NA12878 cell line. The test results showed that a simple consensus approach can significantly improve performance even with a limited number of callers and is more robust and stable than machine learning based ensemble approaches. To fully exploit the multi-callers, we also developed a software package, SomaticCombiner, that can combine multiple callers and integrates a new variant allelic frequency (VAF) adaptive majority voting approach, which can maintain sensitive detection for variants with low VAFs.

## Introduction

Single nucleotide variants (SNVs) and small insertions and deletions (INDELs) are the most frequent and abundant somatic mutations in cancer genomes, and the identification of them is a critical step to understanding cancer genome characterization, clinical genotyping, and treatment^1^. In the past decade, high-throughput next-generation sequencing (NGS) technology has provided opportunities to identify somatic mutations in unprecedented resolution and scale. With the support and evidence of aligned reads, numerous variant callers have been developed to detect somatic variants that are present in tumor samples, but not present in a matched normal sample from the same subjects. Most of these callers use joint-genotype inference from Bayesian models or traditional statistical models combined with specific filters to infer the most likely genotype from allelic counts, (examples include MuSE^2^, JointSNVMix^3^, SomaticSniper^4^, MuTect^5^, LoFreq^6^, Strelka^7^, EBCall^8^ and VarScan^9^). Other callers, such as Snooper^10^ and MutationSeq^11^, extract informative features highly related to variants followed by a classification algorithm to predict variants.

However, it is still very challenging to precisely detect somatic SNVs and INDELs due to low variant allele frequencies (VAFs), sequencing artifacts and lower than desired coverage. Low VAFs in tumor samples are caused by several reasons including tumor-normal cross contaminations, tumor ploidy, sub-clonality (also called intra-tumor heterogeneity), and local copy-number variation in the cancer genome. The performance of a particular caller varies dataset by dataset. Previous studies also showed that the output of different somatic callers for a given dataset is highly divergent and the calling results show a very low level of concordance across callers^12–18^. Due to discrepancies among callers, finding a single best caller for various datasets is considered impractical^18^.

In light of this situation, ensemble approaches have been used to combine prediction results generated by multiple somatic variant callers. The idea^19^ is based on the “wisdom of crowds” the patterns of statistical models used in different classifiers do not necessarily overlap, so the complementary information about these patterns that could potentially be harnessed to improve overall performance. To get a good ensemble, it is generally believed that the base learners should be as accurate as possible, and as diverse as possible^20^. Thus, there are two major questions raised regarding how to construct a feasible and effective ensemble approach. First, how to select a reasonable number of component callers with higher accuracy while maintaining the diversity of the callers^21,22^. Second, how to combine the results from individual callers to determine whether a variant should be called or not. Most ensemble approaches for somatic calling belong to two categories. The simple approach is combining the predictions from multiple callers by simple fixed rules, such as majority voting^19^ or consensus approaches^23,24^. The more complex approaches employ machine learning (ML) methods, which treat prediction results or metrics of individual callers as input features. These inputs are then combined with other genomic features and used to train a classifier that is then applied on an unknown new dataset to predict variants. These ML-based methods include stacking^25^, Bayesian approach^26^, decision trees^18,27^ and deep learning^28,29^. Consensus approaches with a fixed rule are easily implemented and can save tremendous training and prediction time. In contrast to consensus approaches, ML-based ensemble approaches can leverage the information from the training sets with known truth, which may provide potentially better performance than fixed combination rules. However, a downside of the ML-based ensemble approaches is higher computational complexity that may be very sensitive to the training dataset.

Although progress has been made, there are still two significant concerns for current existing ensemble approaches. First, due to insufficient real “ground-truth” somatic variants and evolving software, the caller selection from previous studies may be out of date and not ideal for current studies. A more serious concern is that the replicability and reproducibility of ML-based ensemble methods have not been thoroughly examined. It is well-established that cross-validation provides an optimistic prediction ability compared to the cross-study situation^30^. Most previous ML-based ensemble approaches often relied on cross-validation to illustrate the performance improvement, and while some studies used independent tests, they were very limited. Thus, the previous studies^27,29^ may provide over-optimistic prediction ability because the underlying independently and identically distributed (*i.i.d.*) assumption does not necessarily hold for cross-study datasets. It is known to the field that a number of potential factors in sequencing experiments of different studies may severely impact the generalization of trained predictors. Those factors include but are not limited to the heterogeneity of cancer types, sample collection and quantitation, systematic bias in the sequencing platforms, experimental protocols, sequence coverage, capture kits, and bioinformatics pipelines. The performance of many existing ML models trained from a single training dataset is not necessary to keep consistent performance on other samples.

To address the above questions, there is an emerging need to re-evaluate all current approaches in order to set up a feasible ensemble solution for real applications. To evaluate currently existing callers, we combined several recent real cancer datasets^31–33^, virtual tumor-normal pairs by mixing two Genome in a Bottle Consortium (GIAB) cell lines^34^, with the previous synthetic DREAM datasets^19^. Following the benchmark tests of commonly used single callers, we proposed a VAF adaptive consensus approach to be used for somatic calling. We also implemented the software SomaticCombiner, which can combine up-to-date callers and make prediction decisions to detect somatic SNVs and INDELs. We further evaluated the performance of different ensemble approaches, including majority voting, the new VAF adaptive consensus approach, the deep learning-based caller NeuSomatic^29^, and other commonly used ML approaches. For the ML-based ensemble approaches, we highlighted the performance evaluation on eight WGS datasets using independent unbiased tests.

## Results

### Datasets for evaluation

In order to evaluate the performance of individual callers and ensemble approaches, we started benchmark tests with four synthetic DREAM WGS datasets with the configured mutations generated by a read simulator^19^. In addition to the DREAM datasets, we also introduced four real WGS datasets that cover four different tumor types^31–33^ and reflect more complex variations and artifacts generated from real sequencing reads. These four real datasets have provided curated high-confidence variants, which were derived from high coverage data and different sequencing platforms. Although the completeness of the high-confidence variant sets is unclear, in particular for low VAF sites, we believed that these four real datasets represent the best possible approximations to the ground truth. The summarization of the eight WGS datasets is listed in Table 1. The variant count distributions in different VAF ranges are presented in Supplementary Fig. S1.

**Table 1:** The WGS datasets used in this study. In the “Variant counts” column, “NA” means that high confidence INDELs call sets are not available for the related datasets.

Name	Coverage (normal/tumor)	Variant counts (SNVs/INDELs)
DREAM Set1	27.89/27.89	3,537/NA
DREAM Set2	34.06/34.05	4,332/NA
DREAM Set3	34.23/34.19	7,903/7,991
DREAM Set4	30.02/29.92	16,315/14,230
CLL (chronic lymphocytic leukaemia)	40.90/32.47	1,319/134
AML (acute myeloid leukemia)	126.90/320.91	1,342/NA
COLO (metastatic melanoma)	82.94/92.82	35,543/446
MB (malignant pediatric brain tumor)	271.70/305.62	1,263/347

In addition, we also generated a dilution series deep targeted sequencing dataset experimentally (not in silico), mimicking more realistically the detection of low VAF mutations. Two cell lines from the GIAB project, NA12878 and NA24385, were combined to mimic tumor data. We diluted the NA24385 DNA with an increasing amount of NA12878 DNA from 1 to 50% to estimate the performance in detecting mutations within a range of VAFs. NA24385 was prepared on its own to act like the “normal” data for the tumor-normal paired variant calling of somatic SNVs and INDELs. All samples were subjected to 368 cancer genes as target regions, and we obtained an average on-target coverage of ~ 1,600 × .

As a further step to evaluate the performance on WES datasets, we adopted a public dataset used in the study^24^ and released at PrecisonFDA. Like the deep targeted sequencing data, this dataset was generated from NA11840 with the increasing amount of NA12878 DNA (from 0 to 99.8%) to mimic tumor-normal pairs, and larger exome coding regions were captured. The summarization of the deep targeted sequencing and WES datasets are listed in Table 2.

**Table 2:** The deep targeted sequencing and WES datasets used in this study. Two region sizes are listed in this table. The “calling” regions are used for variant calling. “Comparison” regions inlcude the intersection regions of capture target regions and NA12878 high-confidence regions used for comparison tests.

Name		Mean coverage (“Tumor”)	Mean coverage (“Normal”)	True variant counts in the comparison regions		Region sizes in bases	
				SNVs	INDELs	Calling	Comparison
Deep Targeted Sequencing	NA12878 (1%) vs NA24385	1639.11	1533.77	632	76	2,674,426	2,328,046
	NA12878 (2%) vs NA24385	1642.12					
	NA12878 (5%) vs NA24385	1654.03					
	NA12878 (10%) vs NA24385	1632.73					
	NA12878 (20%) vs NA24385	1693.44					
	NA12878 (50%) vs NA24385	1697.64					
WES	NA12878 (0.5%—100%) vs NA11840	54.38	61.29	9,603	377	45,326,658	44,006,127

Processing details for these datasets can be found in the “Materials and Methods” section.

### Evaluation of individual callers using WGS, deep target sequencing and WES datasets

The goals of this evaluation are two-fold: first, to evaluate the performance of the state-of-arts somatic callers; second, based on the evaluation results, to select callers with the best performance to constitute an ensemble approach for further evaluations.

After initial paper review and screening, we considered only published, freely available, and constantly maintained tools with high citation rates for in-depth evaluations and excluded some callers due to different reasons (e.g., not accept tumor and normal pairs, designed for germline variant calling only, without output in the variant call file (VCF) format, developed for Ion Torrent data, ML-based callers). Thus, we selected eight commonly used somatic callers: LoFreq^6^, MuSE^2^, MuTect^5^, MuTect2, SomaticSniper^4^, Strelka^7^, VarScan^9^, and VarDict^35^.

We adopted three evaluation metrics that are frequently used in the research community to provide comprehensive assessments of imbalanced learning problems, namely, recall (fraction of predicted true variants among all true variants), precision (fractions of predictions that are true among all predicted variants), and *F*1-score (weighted mean of precisions and recall).

**Figure 1:**
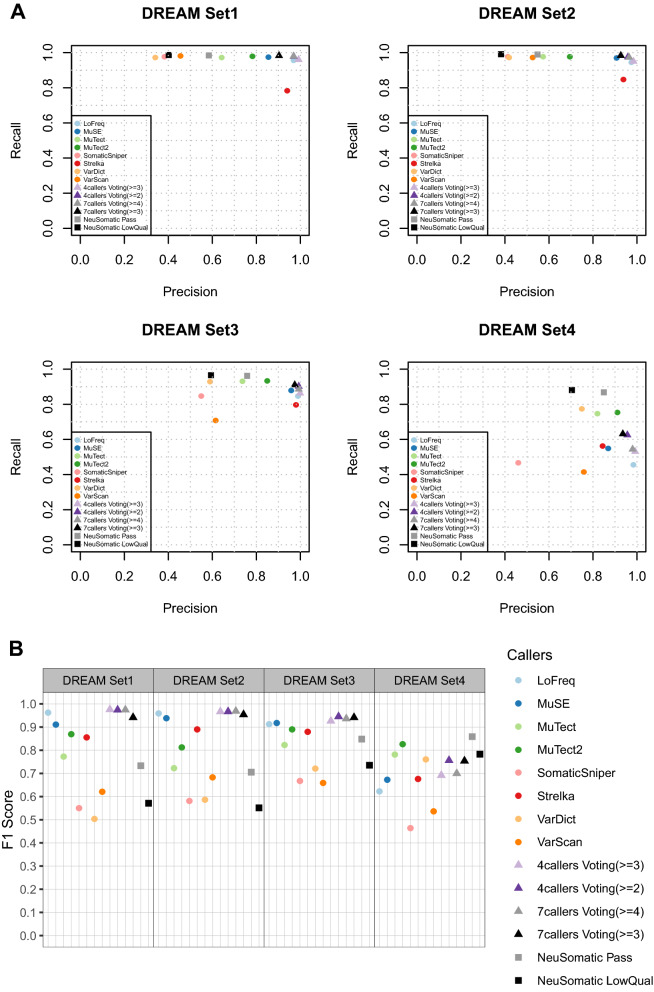
Performance evaluation for somatic SNV calling in the four DREAM WGS datasets. SNVs called by eight individual callers, NeuSomatic, the majority voting with four callers (LoFreq, MuSE, MuTect2 and Strelka) and seven callers (LoFreq, MuSE, MuTect2, SomaticSniper, Strelka, VarDict and VarScan) were compared to the high confidence call sets respectively. Precision, recall and *F*1 scores were calculated for all comparisons and depicted in (**A**) precision-recall plot (**B**) *F*1 plot.

**Figure 2:**
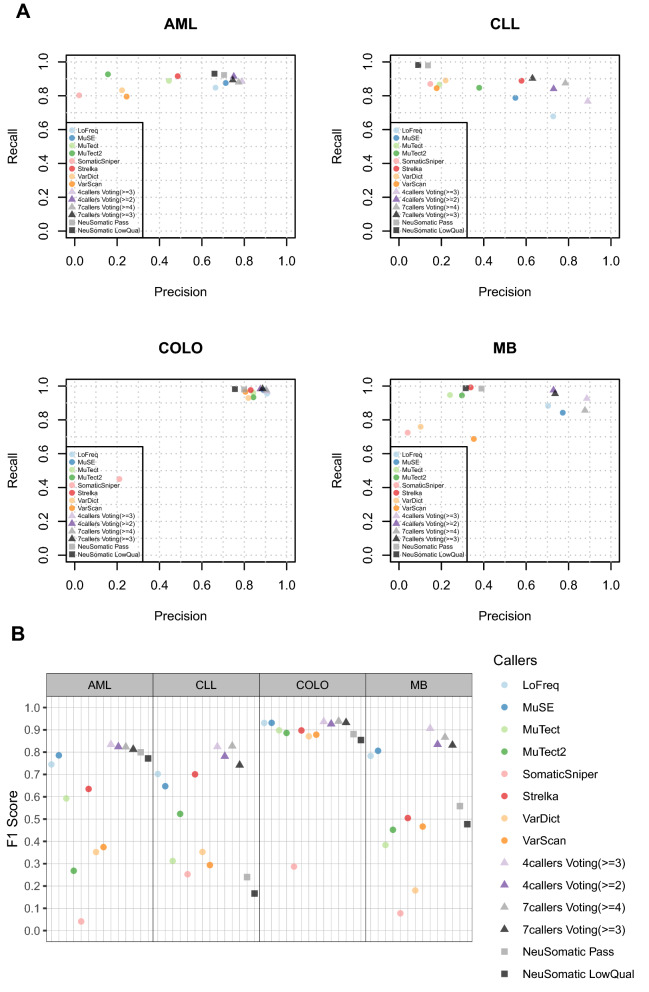
Performance evaluation for somatic SNV calling in the four real WGS datasets. SNVs called by eight individual callers, NeuSomatic, the majority voting with four callers (LoFreq, MuSE, MuTect2 and Strelka) and seven callers (LoFreq, MuSE, MuTect2, SomaticSniper, Strelka, VarDict and VarScan) were compared to the high confidence call sets respectively. Precision, recall and *F*1 scores were calculated for all comparisons and depicted in (**A**) precision-recall plot (**B**) *F*1 plot.

**Figure 3:**
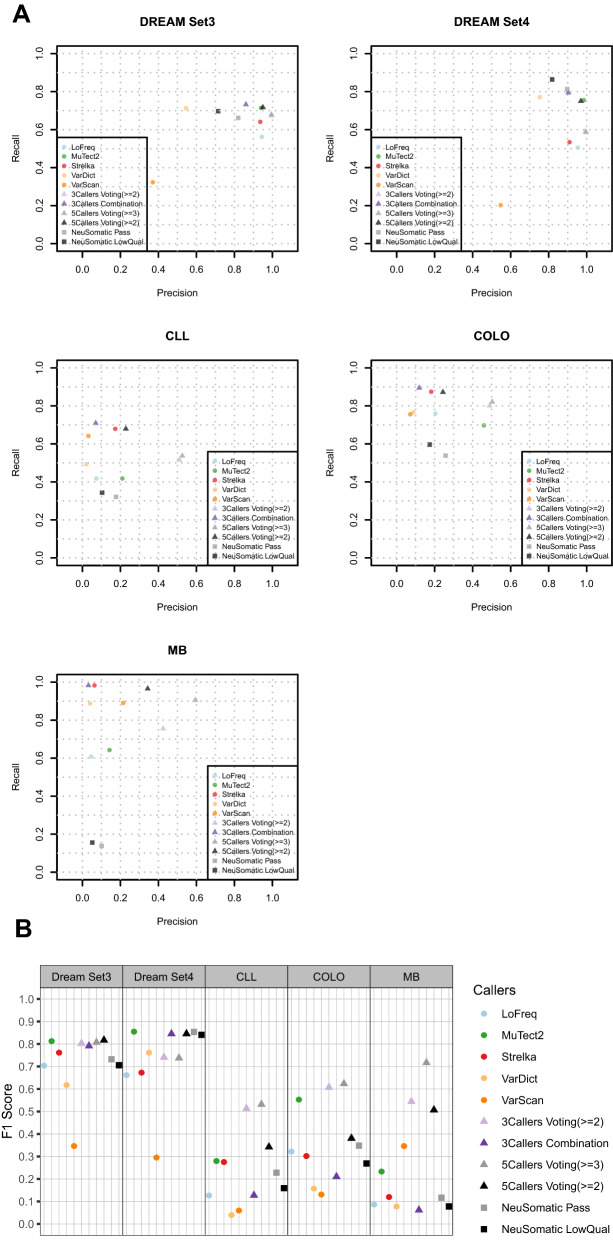
Performance evaluation for somatic INDELs calling in the five WGS datasets. INDELs called by five individual callers, NeuSomatic, combination, or the majority voting with three callers (LoFreq, MuTect2, and Strelka) and the majority voting with five callers (LoFreq, MuTect2, Strelka, VarDict, and VarScan) were compared to the high confidence call sets respectively. Precision, recall and *F*1 scores were calculated for all comparisons and depicted in (**a**) precision-recall plot (**b**) *F*1 plot.

For WGS datasets (Figs. 1, 2 and Supplementary Tables S1–S2), in general, LoFreq, MuSE, and Strelka performed more conservatively, returned fewer SNVs and yielded higher *F*1-scores compared to other callers. MuTect and MuTect2 showed moderate *F*1 scores except for when used for the DREAM set4. VarDict, VarScan and SomaticSniper had lower precision values than other callers, which negatively impacted their overall *F*1 scores. For INDEL calling (Fig. 3), among the five INDEL callers, MuTect2, Strelka and LoFreq showed an overall better performance in terms of *F*1 score when compared to VarDict and VarScan.

**Figure 4:**
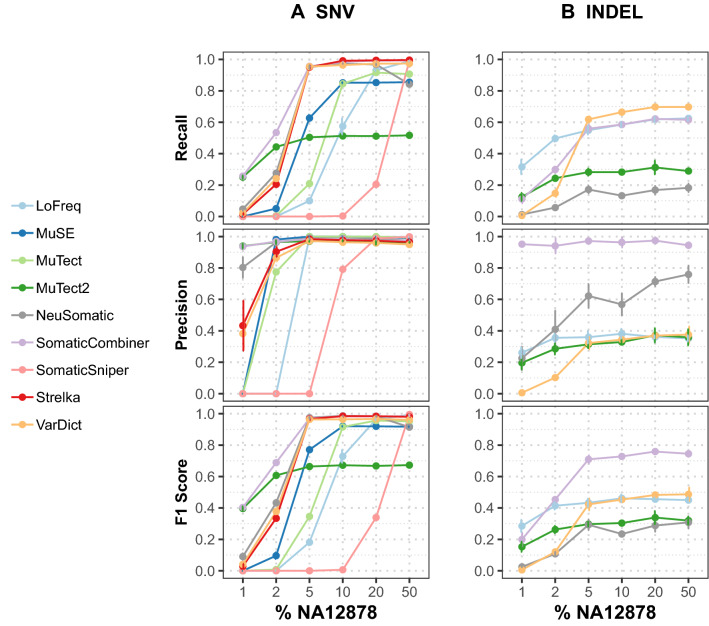
Performance evaluation for somatic calling in the deep sequencing datasets with 1%–50% spiked-in NA12878. Average precision, recall, and *F*1 scores of seven individual callers, NeuSomatic and SomaticCombiner are depicted in (**A**) SNV calling (**B**) INDEL calling. VarScan is not included in this figure because it only called variants in 50% NA12878. For SomaticCombiner, we used the VAF adaptive approach with four callers (LoFreq, MuTect2, Strelka and VarDict) for SNV calling and regular majority voting with three callers (LoFreq, MuTect2 and VarDict) for INDEL calling.

**Figure 5:**
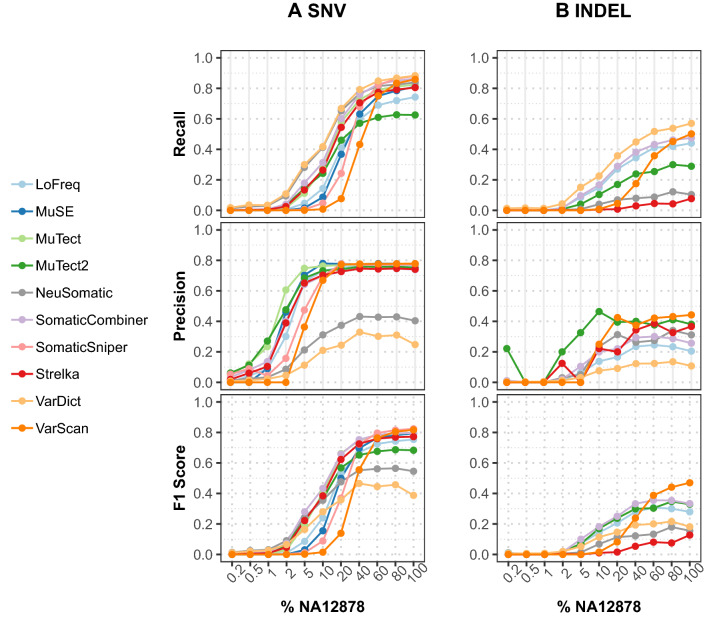
Performance evaluation for somatic calling in the WES datasets with 0.5%–100% spiked-in NA12878. Precision, recall, and *F*1 scores of eight individual callers, NeuSomatic and SomaticCombiner are depicted in (**A**) SNV calling (**B**) INDEL calling. For SomaticCombiner, we used the VAF adaptive approach with four callers (LoFreq, MuTect2, Strelka and VarDict) for SNV calling and regular majority voting with three callers (LoFreq, MuTect2 and VarDict) for INDEL calling.

For the deep sequencing and WES datasets (Figs. 4, 5 and Supplementary Tables S3–S8), Strelka stood out in SNV calling and showed the best overall performance for all samples but displayed low sensitivity in detecting INDELs. LoFreq, MuTect and MuSE showed comparable overall performance across all samples for SNV calling. MuTect2 showed a different tendency than other callers, demonstrating high sensitivity in extremely low VAF samples but lower sensitivity with increasing VAFs. VarDict showed a similar performance to Strelka in deep sequencing data for SNV calling; however, it was very sensitive in WES data with the cost of high false positives. The recall values of the other two callers (VarScan and SomaticSniper) dropped significantly in low VAF sites (< = 20% NA12878 samples). For INDEL calling (Figs. 4B, Fig. 5B, and Supplementary Tables S4, S6 and S8), out of five INDEL callers, only three callers (VarDict, MuTect2, and LoFreq) worked well for all levels.

The full description of the evaluation results for individual callers is presented in Supplemental File 1.

### Availability and implementation of software

Based on the performance evaluation, we implemented a somatic variant combination software SomaticCombiner, that allows users to combine two or more VCFs produced from any seven callers: LoFreq, MuSE, MuTect, MuTect2, Strelka, VarDict, and VarScan. SomaticCombiner is publicly released at https://github.com/mingyi-wang/somatic-combiner. This tool can output a superset of all variants from user-supplied callers in a VCF, add the callers' decisions and tumor VAF values in the “INFO” column. With the input option, users can set a minimal tumor VAF threshold to select desired variants.

From our benchmark tests, in addition to the simple majority voting method, we also derived a VAF adaptive voting approach to further tune the decision making. The idea is based on observations that two callers, Strelka and MuTect2, detected SNVs in low VAF sites with high precision values in deep targeted sequencing data (Fig. 4A). To avoid missing those low VAF SNVs in a regular majority voting step, the new voting approach flag SNVs as “PASS” if one of the three following conditions are met:

- if called by Strelka and MuTect2 are called and tumor VAF >  = 0.03 and tumor VAF <  = 0. 1, then flag “PASS”;
- if tumor VAF < 0.03 and called by MuTect2, then flag “PASS”;
- if called by >  = 50% of callers, then flag “PASS”.

With this approach, we tuned the confidence cutoff to reflect the fact that only very few callers can detect low or ultra-low VAF SNVs. Based on both of the majority voting (called by >  = 50% of callers) and above VAF adaptive voting approaches, SomaticCombiner further tags the confidence levels in the “FILTER” column for each variant in the output VCF.

### Evaluation of our consensus approach

For the ensemble evaluations, we first investigated whether a simple majority voting process can improve the performance in WGS data. For SNV calling, we used two caller sets with stringent cutoffs in each: one used four callers (LoFreq, MuSE, MuTect2 and Strelka) with called by (> = 2) and (> = 3) callers as two cutoffs; the other used seven callers (LoFreq, MuSE, MuTect2, SomaticSniper, Strelka, VarDict and VarScan) with cutoffs when called by (> = 3) and (> = 4) callers. We also included a newly reported deep learning-based ensemble method NeuSomatic^29^ in this benchmark. Note that in the NeuSomatic ensemble mode, we followed its usage guidance and combined the prediction results from six callers (Mutect2, VarScan, SomaticSniper, VarDict, MuSE and Strelka), which also means more information from extra callers were used compared to the four callers in the majority voting.

For the SNV calling, out of eight WGS datasets (Figs. 1, 2 and Supplementary Table S1), the majority voting approach performed consistently better than any individual callers and NeuSomatic in seven datasets. However, NeuSomatic performed poorly across all datasets, even worse than individual callers in most of the cases. The only exception is the DREAM set4, where NeuSomatic + Pass yielded the highest *F*1 score (0.859) in this dataset, and both MuTect and MuTect2 also performed better than the majority voting approach. This result indicates that the deep learning model trained by the DREAM set3 works well on set4. However, NeuSomatic’s inconsistent performance on other datasets showed the ML-based approach has a high dependency on training datasets.

In most cases, the four-callers voting approach performed comparably to seven-callers, suggesting that we can exclude the other three callers and maintain similar or even better performance while reducing the computational burden.

For the INDEL calling (Fig. 3 and Supplementary Table S2) in WGS data, we evaluated four majority voting settings and NeuSomatic. Four settings include three callers (LoFreq, MuTect2, Strelka) with two stringent cutoffs (the combination of all three callers and called by >  = 2 callers) versus all five callers (LoFreq, MuTect2, Strelka, VarDict, and VarScan) with two cutoffs (called by >  = 2 and >  = 3 callers). The majority voting approach still outperformed NeuSomatic and all individual callers in four datasets in terms of *F*1 score except for the DREAM set4 (Fig. 3B and Supplementary Table S2). In the DREAM set4, MuTect2 showed the best *F*1 score (0.855), followed by NeuSomatic + Pass (0.853) and the majority voting (0.846). Unlike with SNV calling, the performance of the majority voting approach when paired with a more stringent cutoff dramatically increased *F*1 scores, which suggested that false positives are a more severe issue for INDEL calling.

For both deep targeted sequencing and WES datasets, we applied a four-caller ensemble with our own VAF adaptive consensus approach, which consisted of four callers for SNV calling: LoFreq, MuTect2, Strelka and VarDict. For INDEL calling, we used the regular majority voting with three callers: LoFreq, MuTect2, and VarDict. Unlike the WGS data, we used VarDict instead of MuSE in SNV calling and instead of Strelka in INDEL calling due to the previous benchmark results.

For the deep targeted sequencing data (Fig. 4A and Supplementary Table S3), we observed that SomaticCombiner with the VAF adaptive consensus approach achieved the best overall performance on SNV calling. It performed better than all individual callers in three levels 1%, 2%, and 5% NA12878 and better than NeuSomatic in all levels except 10% and 20% NA12878. The *F*1 score was only slightly lower than SomaticSniper and LoFreq in 50% NA12878 and Strelka in 20% and 10% NA12878 but still achieved second-best performance in 10%, 20% and 50%. For INDEL calling (Fig. 4B and Supplementary Table S4), it is noteworthy that majority voting with three callers improved performance significantly at all purity levels; only performed behind LoFreq at the 1% NA12878 purity level.

For the WES data (Fig. 5A and Supplementary Table S7), our ensemble approach also improved overall SNV calling performance and was robust in all purity levels. The *F*1 scores of our ensemble (0.797, 0.794) are only slightly lower than SomaticSniper (0.823, 0.815) and VarScan (0.817, 0.805) in 100% and 80% levels; the *F*1 scores (0.003, 0.009, 0.014) were also lower than VarDict (0.011, 0.020, 0.027) at very low purity levels 0.2%—1%. However, SomaticCombiner performed better than all other callers at all other purity levels. SomaticCombiner performed better than NeuSomatic in 2%–100% NA12878 purity levels while had lower recall values than NeuSomatic in very low 0.2%–1% NA12878 levels. For INDEL calling (Fig. 5B and Supplementary Table S8), we also see the majority voting with three callers improved overall performance and achieved the best performance between 2%—40% and second-best performance in 60%—100%.

### Evaluation of other ML-based ensemble approaches

We investigated whether other ML-based ensemble methods could further improve performance because we observed improvement using the consensus approach. We used independent tests rather than cross-validation to avoid over-fit and bias from a single training dataset.

#### Feature selection

**Figure 6:**
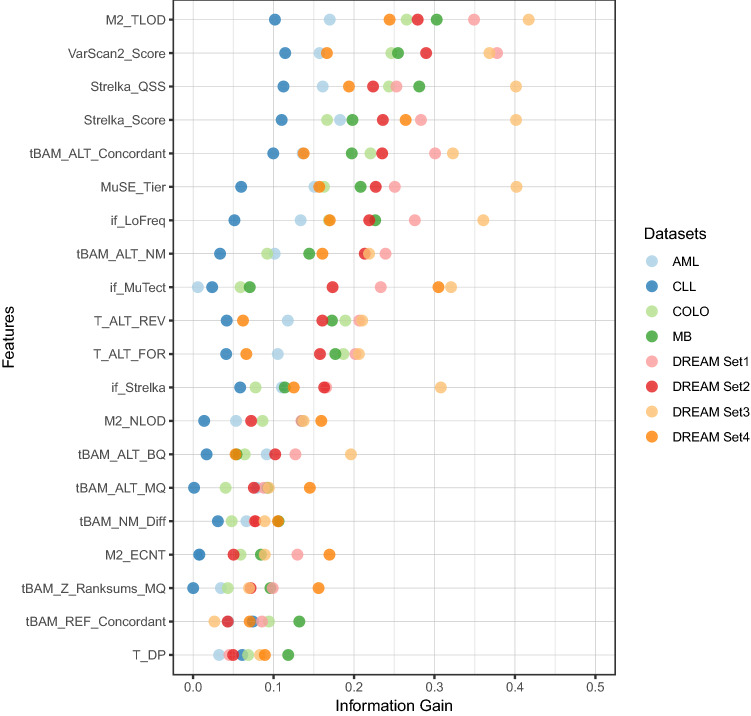
Top 20 genomic features or metrics selected by information gain (IG) values and eight WGS datasets. The details of the features are described in Supplementary Table S7.

For ML-based ensemble approaches, we needed to select important features that are relevant to true or false somatic variant classification. Based on 100 original features introduced by a previous study^27^, we employed information gain (IG) as the measurement to further select important features that best discriminate between the two classes. For SNV classification, we summed up the 100 IG values calculated from all eight training datasets and then sorted them according to the IG sums. The top 20 ranked features are listed in Fig. 6 and their definitions are given in Supplementary Table S9.

We observed that the most significant features are mainly consistent among the different datasets (Fig. 6). Interestingly, more than half of the top-ranked features are metrics produced from the somatic callers themselves (Supplementary Table S9), e.g., MuTect2 TLOD value, the Phred-scale *p* values from Fisher’s exact test (used as VarScan2 score), and Strelka QSS. The feature selection results illustrated that somatic callers not only predict variants but provide valuable measurements related to somatic variant prediction as well. This also suggested that ML-based ensemble approaches are more powerful than ML-based callers from pure genomic features (such as Snooper^10^ and MutationSeq^11^) since metric values produced from callers are incorporated. The remaining ten genomic features are mainly extracted from tumor BAMs, and most of them are related to ALT reads, such as depth, mapping quality, and base quality of ALT reads.

For ML tests, in each training dataset, the top 20 features were selected for training and predictions in the next step.

#### ML tests

To ensure the comparisons were comprehensive, we included four widely used classifiers as alternative learning approaches: Support Vector Machine (SVM), *k*-Nearest Neighbors (*k*-NN), naïve Bayesian (NB) and Random Forests (RF) and trained them using eight WGS datasets.

**Figure 7:**
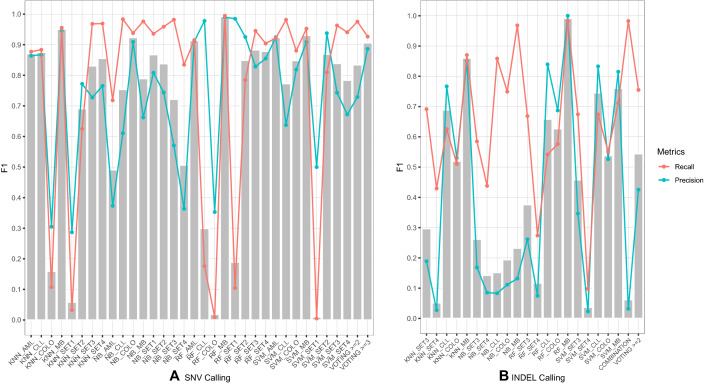
Comparison of performance between the ML-based approaches and the majority voting approach in the MB dataset. (**A**) SNV calling (**B**) INDEL calling. We used the regular majority voting with four callers (LoFreq, MuSE, MuTect2 and Strelka) for SNV calling and three callers (LoFreq, MuTect2 and Strelka) for INDEL calling.

The performance comparisons of ML-based methods versus the majority voting approach are listed in Fig. 7 and Supplementary Figs. S2–S12. From the results, we can see that ML-based methods showed high variance across the different models and training sets. For example, in the MB dataset (Fig. 7A), the *F*1 scores are varied from the lowest 0.019 (RF + COLO) to the highest 0.924 (NB + COLO). Out of all 28 realistic models with four classifiers and seven training datasets other than itself, only three ML models (NB + COLO: 0.924, RF + AML: 0.913 and SVM + AML: 0.923) performed slightly better than the majority voting (> = 3) (0.906). The performance improvement attributed to increased precision values, which are 0.909, 0.911 and 0.925, respectively, compared to 0.886 from the majority voting. For both SNV and INDEL calling in all other datasets, we can see the same trend (Fig. 7B and Supplementary Fig. S2–S12). In summary, although the ML-based approaches can potentially further improve performance, the poor generalization performance across all datasets indicates that ML-based approaches are not necessarily applicable.

We also observed that the models trained from the DREAM set3 improved prediction accuracy on the DREAM set4 (Supplementary Figs. S5 and S10). For SNV calling (Supplementary Fig. S5), three classifiers trained from the DREAM set3 achieved better *F*1 scores (*k*-NN: 0.805, RF: 0.821, and SVM: 0.813) than the majority voting approach (> = 2) (0.756) and NB (0.751) is only slightly lower than the voting approach. For INDEL calling (Supplementary Fig. S10), all four classifiers (*k*-NN: 0.865; NB: 0.847; RF: 0.864; SVM: 0.856) performed better than the majority voting (> = 1) (0.846). This result is consistent with the better performance obtained from NeuSomatic on the DREAM set4 using the model trained by the DREAM set3. This suggests that the DREAM set3 and set4 may have a minimal variance in 20 feature vectors because both were generated from the same simulator. To compare relatedness among different datasets, we randomly selected 1,000 true SNVs from each dataset with 20 features listed in Fig. 6 and then performed the principal component analysis (PCA) on those datasets. The PCA plot (Supplementary Fig. S13) showed the SNVs selected from DREAM set3 and set4 are closer with each other when compared to other datasets, which partially explained the reproducibility of ML-based ensembles from these two DREAM datasets.

## Discussion

We have performed benchmark tests using the WGS, WES, and deep targeted sequencing datasets, and our results can serve as a reference for the research community to select appropriate somatic callers and optimize caller combination(s). From performance comparison results of individual callers, we observed that there is no clear winner for all datasets, and each caller has its own strengths and weaknesses in different settings. The varied performance of callers indicates that using ensemble approaches to combine them is essential for performance improvement.

As one ensemble strategy, ML-based approaches are more advanced models that can utilize feature information learned from training datasets. However, from our cross-study independent tests, we saw that the performance improvement of an ML model highly depends on the training datasets and classifiers; in most cases, ML models performed even worse than individual callers without ensemble. ML-based ensemble methods are far from stable and robust due to constraints on the training sets. Our tests showed that the deep learning-based model also faces similar issues. Therefore, we caution the use of existing trained ML models and recommend users carefully examine the performance of prediction results to decide whether they are acceptable for their own applications.

Currently, the lack of enough datasets with ground truth hinders us from adequately addressing the reproducibility issue of ML-based ensemble methods. During this study, we have also explored two strategies to set up a training dataset to mitigate the issue of insufficient training datasets and data heterogeneity. One is more straightforward, we combined all the training sets and thus ignored heterogeneity to generate a large harmonized training set. Another approach is using a testing dataset itself to generate a pseudo training dataset; in this approach, we treat all variants called by all callers as high confidence variants and use them as true positives, and take remaining variants called by one caller only as true negatives, then run the training process. However, both training approaches are not satisfactory and cannot improve overall performance versus the majority voting approach. From DREAM set 3 to improve DREAM set4, we can find that the training set is more critical than ML model selection because ML ensemble approaches almost always enhance performance in all classifiers. Thus, a future direction may be building a statistical framework to measure whether a new study is similar enough to existing training sets to improve the generalization ability of a trained ML model.

In contrast to ML-based ensemble methods with unsolved training problems, the consensus ensemble approach is more straightforward and does not need training. The results show that the consensus approach is robust and can considerably increase overall accuracies across all datasets with different backgrounds, even using a limited number of callers. To facilitate the usage of consensus ensemble approaches, we developed a software package, SomaticCombiner, which provides the flexibility to combine any two or more somatic callers to exploit the power of the ensemble. In addition to the regular majority voting approach, we also proposed the VAF adaptive consensus approach, which can maintain detective sensitivity for low VAF SNVs. Low VAF SNVs are challenging to distinguish from artifacts and most callers have a difficult time detecting them. Thus, this new approach can prevent low VAF SNVs from being rejected by a hard cutoff used in the regular majority voting approach. This property is of crucial importance for studying cancer subclones and tumor samples with genetic intra-heterogeneity.

For the deep sequencing and WES data, this VAF adaptive consensus approach always outperformed the majority voting approach in SNV calling when tumor purity was below 20% and expected heterozygous variants with VAFs < 10%. At 1%–5% tumor purity levels, especially for higher coverage (≥ 500 ×) tumor samples, this approach performed better than the majority voting approach. For moderate or higher VAFs (≥ 10%), the results had slightly lower *F*1 scores compared to the majority voting approach due to increased false positives. We have tested this VAF adaptive consensus approach in WGS data as well, but we found that this approach is only better than the majority voting in DREAM set4 and COLO among all eight WGS datasets. It is easy to understand that the VAF adaptive consensus approach results in more false positives than the regular majority voting approach. On the other hand, unlike NA12878 samples that can spike in low VAF variants, the current real WGS datasets still lack enough true low VAFs variants for validation at this stage. For example, among four synthetic DREAM datasets, set1-set3 are low complexity and all VAFs of true variants are higher than 10%; only the DREAM set4 includes very few low VAF (< 10%) variants. For real WGS datasets, in both CLL and MB datasets^31^ , VAF < 2% were deemed a call unreliable and difficult to be confidently verified, and thus these two datasets also lack low VAF SNVs for evaluation. Although there are some limitations for evaluation, the VAF adaptive consensus approach is a promising alternative method for the detection of low VAF mutations.

As a tradeoff for performance improvement and the cost of running more callers, we showed that a feasible solution could be found with the consensus approach using only three or four callers in an ensemble. Our tests have demonstrated that two ensemble settings can improve performance for somatic SNV calling, i.e., the VAF adaptive consensus approach with four callers (LoFreq, MuTect2, Strelka and VarDict) for deep targeted sequencing and WES data and the regular majority voting with four callers (LoFreq, MuSE, MuTect2 and Strelka) for WGS datasets. For INDEL calling, we recommend using the majority voting with three callers (LoFreq, MuTect2 and VarDict) for deep targeted sequencing and WES data and three callers (LoFreq, MuTect2 and Strelka) for WGS data. Although the above consensus approach achieved success in this study, we were still unable to propose a universal caller combination and a fixed combination rule as a best practice for all cases due to various datasets. As we observed from benchmark tests, several factors have a substantial impact on the performance of somatic callers, such as coverage of depth, tumor purity, allelic frequency, and capture regions. Thus, a better understanding of sequencing data and research goals is still needed before customizing an ensemble for a somatic calling study.

In this study, we only focused on somatic mutation calling part of the entire analytical process. Therefore, the upstream and downstream steps (such as alignment approaches, base quality recalibration, and post-calling filtering) were fixed. Their impact on the somatic variant calling is out of the scope of this paper. For low VAF detection, further investigation is recommended on how to fine-tune the threshold on genomic metrics in post-calling filtering steps.

## Materials and methods

### Data collection

We downloaded the BAM files for synthetic data set1, set2, set3, and set4 from ICGC-TCGA DREAM Mutation Calling challenge (https://www.synapse.org/#!Synapse:syn312572/wiki). The four DREAM datasets show different complexity levels with set1 being simplest and set4 being the most complex: set1 and set3 with 100% tumor cellularity; set2 and set4 with 80% cellularity and set3 and set4 with subclonality. For the DREAM datasets, we also downloaded the somatic truth sets from https://www.synapse.org/#!Synapse:syn2177211.

We further collected four real WGS data sets with the high-quality somatic variant call sets. These datasets include tumor samples from chronic lymphocytic leukemia (CLL) and a malignant pediatric brain tumor in the cerebellum (MB)^31^, acute myeloid leukemia (AML)^32^, and metastatic melanoma cell line (COLO829)^33^.

Both the AML and COLO829 datasets were downloaded from dbGAP with accession IDs phs000159 and phs000932. For the COLO829 dataset, we selected one Illumina pair with the Run IDs: SRR3184219 and SRR3184215. For the AML dataset, we used the sample pair with the Run IDs: SRR2470200 and SRR2177258. The FASTQs of the CLL and MB datasets were downloaded from the European Bioinformatics Institute https://www.ebi.ac.uk/ega/datasets/EGAD00001001858 and https://www.ebi.ac.uk/ega/datasets/EGAD00001001859, respectively. For four real WGS datasets, the curated high-confidence somatic variant call sets were downloaded from the related supplementary files of the studies^31–33^. For the AML dataset, the “platinum” somatic variant list that contained high-quality validated sites was used for evaluation.

The raw FASTQ files of NA12878-NA11840 dilution WES series datasets^24^ were downloaded from https://precision.fda.gov/ (go to Files > Explore > Added by: ‘maurizio.callari’).

### Deep target sequence

#### DNA preparation

DNA samples from two unrelated NIST GIAB reference samples (NA12878, NA24385) were ordered from Coriell Institute. For each sample, DNA was quantified using Quant-iT PicoGreen dsDNA Reagent (Life Technologies, ThermoFisher Scientific, Waltham, MA). For each library prepared, a total of 200 ng genomic DNA was aliquoted. Libraries were prepared using the following proportions (by mass) of the two GIAB samples: NA24385 alone (to be used the “normal” data for tumor/normal variant calling), and NA12878 spiked into NA24385 at 1:99, 2:98, 5:95, 10:90, 20:80, and 50:50 (combinations to be used as the “tumor” data for tumor/normal variant calling). Each of these combinations was replicated, for a total of 14 libraries. The expected VAF of heterozygous variants in the “tumor” was 0.5%, 1%, 2.5%, 5%, 10%, and 25%, depending on the proportions of each sample used. To prepare sequencing libraries of each 200 ng aliquot, the DNA was first purified using Agencourt AMPure XP Reagent (Beckman Coulter Inc, Brea, CA) according to manufacturer's protocol. An adapter-ligated library was prepared with the KAPA HyperPlus Kit (KAPA Biosystems, Wilmington, MA) using IDT's xGen Dual Index UMI Adapters (IDT, Coralville, IA) according to KAPA-provided protocol.

#### Pre-Hybridization LM-PCR

The deep target captured sequencing was performed at the NCI Cancer Genomics Research Laboratory (CGR) as previously described^36^. Sample libraries were amplified pre-hybridization by ligation-mediated PCR consisting of one reaction containing 20 μL library DNA, 25 μL 2 × KAPA HiFi HotStart ReadyMix, and 5μL 10 × Library Amplification Primer Mix (which includes two primers whose sequences are: 5′-AATGATACGGCGACCACCGA-3′ and 5′-CAAGCAGAAGACGGCATACGA-3′). PCR cycling conditions were as follows: 98 °C for 45 s, followed by 5 cycles of 98 °C for 15 s, 60 °C for 30 s, and 72 °C for 30 s. The last step was an extension at 72 °C for 1 min. The reaction was kept at 4 °C until further processing. The amplified material was cleaned with Agencourt AMPure XP Reagent (Beckman Coulter Inc, Brea, CA) according to the KAPA-provided protocol. Amplified sample libraries were quantified using Quant-iT PicoGreen dsDNA Reagent (Life Technologies, ThermoFisher Scientific, Waltham, MA).

#### Liquid phase sequence capture

Prior to hybridization, amplified sample libraries with unique barcoded adapters were combined in equal amounts into 1.1 μg pools for multiplex sequence capture. Exome sequence capture was performed with a custom NimbleGen SeqCap EZ Choice Library, with 2.6 Mb of genomic sequence targeted across 368 genes (Roche NimbleGen, Inc., Madison, WI, USA). Prior to hybridization, the following components were added to the 1.1 μg pooled sample library, 2 μL of xGen Universal Blocker-TS (IDT, Coralville, IA), and 5 μL of 1 mg/mL COT-1 DNA (Invitrogen, Inc., ThermoFisher Scientific, Waltham, MA). Samples were dried down by puncturing a hole in the plate seal and processing in an Eppendorf 5,301 Vacuum Concentrator (Eppendorf, Hauppauge, NY, USA) set to 60 °C for approximately 1 h. To each dried pool, 7.5 μL of NimbleGen Hybridization Buffer and 3.0 μL of NimbleGen Hybridization Component A were added and placed in a heating block for 10 min at 95 °C. The mixture was then transferred to 4.5 μL of EZ Choice Probe Library and hybridized at 47 °C for 64 to 72 h. Washing and recovery of captured DNA were performed as described in NimbleGen SeqCap EZ Library SR Protocol^36^.

#### Post-hybridization LM-PCR

Pools of captured DNA were amplified by ligation-mediated PCR consisting of one reaction for each pool containing 20 μl captured library DNA, 25 μL 2 × KAPA HiFi HotStart ReadyMix, and 5 μL 10 × Library Amplification Primer Mix. PCR cycling conditions were as follows: 98 °C for 45 s, followed by 8 cycles of 98 °C for 15 s, 60 °C for 30 s, and 72 °C for 30 s. The last step was an extension at 72 °C for 1 min. The reaction was kept at 4 °C until further processing. The amplified material was cleaned with Agencourt AMPure XP Reagent (Beckman Coulter Inc, Brea, CA), according to NimbleGen SeqCap EZ Library SR Protocol. Pools of amplified captured DNA were then quantified via Kapa's Library Quantification Kit for Illumina (Kapa Biosystems, Wilmington, MA) on the LightCycler 480 (Roche, Indianapolis, IN)^36^.

#### Sequencing

The resulting post-capture multiplexed sequencing libraries were sequenced on an Illumina NovaSeq 6,000 following Illumina-provided protocols for 2 × 150 bp on an S2 flowcell (Illumina, San Diego, CA), using dual index sequencing. Libraries were sequenced to a depth of approximately 5,000 × coverage prior to deduplication.

### Sequencing data analysis

#### Primary analysis

Details of the primary analysis pipeline have been previously published^37^. For the CLL, MB, deep targeted sequencing, and WES datasets, FASTQ files were first trimmed using the Trimmomatic program^38^(v0.36), which marks all low-quality stretches (average quality score < Q15 in a 4-bp sliding window) and reports the longest high-quality stretch of each read. Only read pairs with both ends no shorter than 36 bp were used. For CLL and MB datasets, FASTQ files from different labs were concatenated with the same samples. Reads were then aligned to the hg19 reference genome using the Novoalign software (v3.00.05) (https://www.novocraft.com). Duplicate reads due to either optical or PCR artifacts were removed from further analysis using the MarkDuplicates module of the Picard software (v1.126; https://picard.sourceforge.net/). Additionally, our analysis uses only properly aligned read pairs, in the sense that the two ends of each pair must be mapped to the reference genome in complementary directions and must reflect a reasonable fragment length (300 ± 100 bp). The high-quality alignments for each sample were further refined according to a local realignment strategy around known and novel sites of insertion and deletion polymorphisms using the RealignerTargetCreator, IndelRealigner and BQSR modules from the Genome Analysis Toolkit (GATK v3.8.1). Thus, the BAMs files were ready for somatic calling. For the DREAM, AML and COLO datasets, we used the downloaded BAMs for somatic calling.

#### Somatic variant calling

For somatic calling, we used MuTect v1.1.7, MuTect2 (GATK v3.7–0), MuSE v1. 0rc, Strelka v2.7.1, LoFreq v2.1.3.1, VarDict v1.6.0, VarScan v2.3.9 and SomaticSniper v1.0.5.0. For most of the callers, we used default settings or followed the usage instructions from authors. For somatic calling with MuTect and MuTect2, we supplied dbSNP v138 and COSMIC v80 in WGS datasets. We skipped these two options for deep targeted sequencing and WES datasets because the NA12878 germline sample was used for simulating the tumor sample. For MuSE, we used “-G” option for WGS and “-E” for deep sequencing data, respectively. For VarScan, we followed the somatic calling postprocessing. For VarDict WGS calling, we utilized the bcbio-nextgen v 1.1.5 (https://github.com/bcbio/bcbio-nextgen) package because it was well fine-tuned when it was integrated with this package and yields better performance than standalone VarDict calling (Both results are listed in Supplementary Tables S1 and S2). For deep sequencing and WES data, we used VarDict standalone package because we did not see the performance improvement from Bcbio. We followed the two parameters in the pipe command line “var2vcf_paired.pl -P 0.9 -m 4.25 -f 0.01 -M” and filtered with “((AF*DP < 6) && ((MQ < 55.0 && NM > 1.0) || (MQ < 60.0 && NM > 2.0) || (DP < 10) || (QUAL < 45)))” using GATK VariantFiltration module according to the setting used by Bcbio.

For NeuSomatic calling, we followed the Ensemble mode and used the steps suggested by NeuSomatic github. At first, we generated Ensemble.tsv using SomaticSeq.Wrapper.sh (downloaded from https://github.com/bioinform/somaticseq) and six callers VCFs (MuTect2, MuSE, Strelka, SomaticSniper, VarDict, and VarScan). Then, NeuSomatic v0.2.1 with NeuSomatic_v0.1.3_ensemble_DREAM3.pth was used for prediction on WGS datasets.

#### Variant calling comparisons

For somatic variant evaluations in WGS datasets, we used evaluator.py (https://github.com/Sage-Bionetworks/ICGC-TCGA-DREAM-Mutation-Calling-challenge-tools/blob/master/evaluator.py) for comparisons. The NA12878 and NA24385 high confidence call sets were downloaded from ftp://ftp-trace.ncbi.nlm.nih.gov/giab/ftp/release/NA12878_HG001/NISTv3.3.2/GRCh37/ and ftp://ftp-trace.ncbi.nlm.nih.gov/giab/ftp/release/AshkenazimTrio/HG002_NA24385_son/NISTv3.3.2/GRCh37/. For deep sequencing data, we removed NA24385 variants from NA12878 high confidence call sets as the final reference call sets for comparison purposes. For WES NA12878 spiked-in datasets, we used the truth sets generated by authors^24^ and downloaded from https://precision.fda.gov/. We used vt v0.5772^39^ for INDEL normalization before INDEL comparisons. For both deep sequencing and WES data, we used USeq_8.9.6 VCFComparator; only the variants in the intersection regions of GIAB NA12878 high confidence regions and capture target regions were used for comparisons.

For calculation of comparison measurements, we defined the number of variants reported in the high-confidence or truth call sets as true variants and the detected variants as positive.

- Recall = number of detected true variants / number of true variants.
- Precision = number of detected true variants /number of detected variants.
- False discovery rate (FDR) = 1 − Precision.
- False negative rate (FNR) = 1 − Recall.
- *F*1-score = 2 × Precision × Recall / (Precision + Recall).

#### Machine learning approaches

As full independent tests, for each WGS dataset, we used a union of call sets generated from multiple callers with true or false somatic variants as classification labels and ran feature selection with IG. Then, we generated a training dataset with 20 feature values as input for supervised learning. We repeated such a training process for all eight WGS datasets with the above four classifiers. Thus, 32 trained SNV models (four classifiers trained from eight datasets) and 20 INDEL models (five datasets with four classifiers), which fit training sets were produced. We applied these trained models to each WGS dataset as an unseen dataset and ran independent classification tests. Notice that for each test dataset, there are four models trained by the test dataset itself. In practice, it is impossible to run training and testing using the same dataset, so we used this result only to show the best attainable performance. To avoid extra information used for ML models and be fair in comparisons with the majority voting, we used the same caller sets for ML-based learning, i.e., four callers (LoFreq, MuSE, MuTect2 and Strelka) for SNV calling and three callers (LoFreq, MuTect2 and Strelka) for INDEL calling. For each dataset, we employed SomaticSeq.Wrapper.sh provided by SomaticSeq^27^ (https://github.com/bioinform/somaticseq) to generate Ensemble.sSNV.tsv and Ensemble.sINDEL.tsv to run machine learning training and tests. Because some true variants were missed by all callers and not reported in Ensemble.tsv, we also counted those missing variants and then corrected the final recall, precision, and *F*1 values for comparisons using R scripts. Before running ML-based training and test, the normalization step to rescale the values between 0 and 1 for 20-feature values in the datasets was performed.

Several R packages (FSelector, e1071, class, gmodels, randomForest, ggfortify) were used for feature selection, machine learning tests and PCA analysis. In our tests, SVMs with a linear kernel and 3-NN were applied in the ML tests. Software development.

The SomaticCombiner software was developed by Java and GATK htsjdk v2.8.1 API (https://samtools.github.io/htsjdk/javadoc/htsjdk/). The htsjdk API was employed to implement low-level VCF file parsing and output. In this software, we used a Hash table to implement variant search and merge, which can scale up to large VCFs and speed up running time. In the merge step, the original annotations or genotype metrics values returned from individual callers are integrated into the final output VCF file. In order to implement the VAF adaptive consensus approach, the tumor allelic frequency for each variant is extracted from the VCFs generated from individual callers based on the configured callers’ priority.

## Supplementary information

Supplementary Figures.

Supplementary information.

Supplementary Tables.

## Data Availability

Biological sequencing data is available from NCBI Sequence Read Archive under the BioProject accession number: PRJNA636886.
