# Supplementary material for: SomaticCombiner: improving the performance of somatic variant calling based on evaluation tests and a consensus approach
Source: Sci Rep. 2020 Jul 30;10:12898. doi: 10.1038/s41598-020-69772-8 (PMC7393490; doi:10.1038/s41598-020-69772-8)
Supplement: Supplementary file 1 — Supplementary Figures. [file 41598_2020_69772_MOESM1_ESM.pdf]

Supplementary Figure Legends

Figure S1. Relative frequencies of SNV and INDEL counts in different tumor VAF ranges.

Figure S2-S8. The performance comparisons for SNV calling between ML-based approaches and majority voting approach with four callers (LoFreq, MuSE, MuTect2 and Strelka) in the DREAM set1-set4, AML, CLL and COLO datasets.

Figure S9-S12. The performance comparisons for INDEL calling between ML-based approaches, the combination of three INDEL callers (LoFreq, MuTect2 and Strelka) and majority voting approach in the DREAM set3, DREAM set4, CLL and COLO datasets.

Figure S13. PCA analysis plot for eight WGS datasets. PCA analysis was performed on 1000 randomly selected true SNVs from each WGS dataset with their top 20 features.

Figure S1

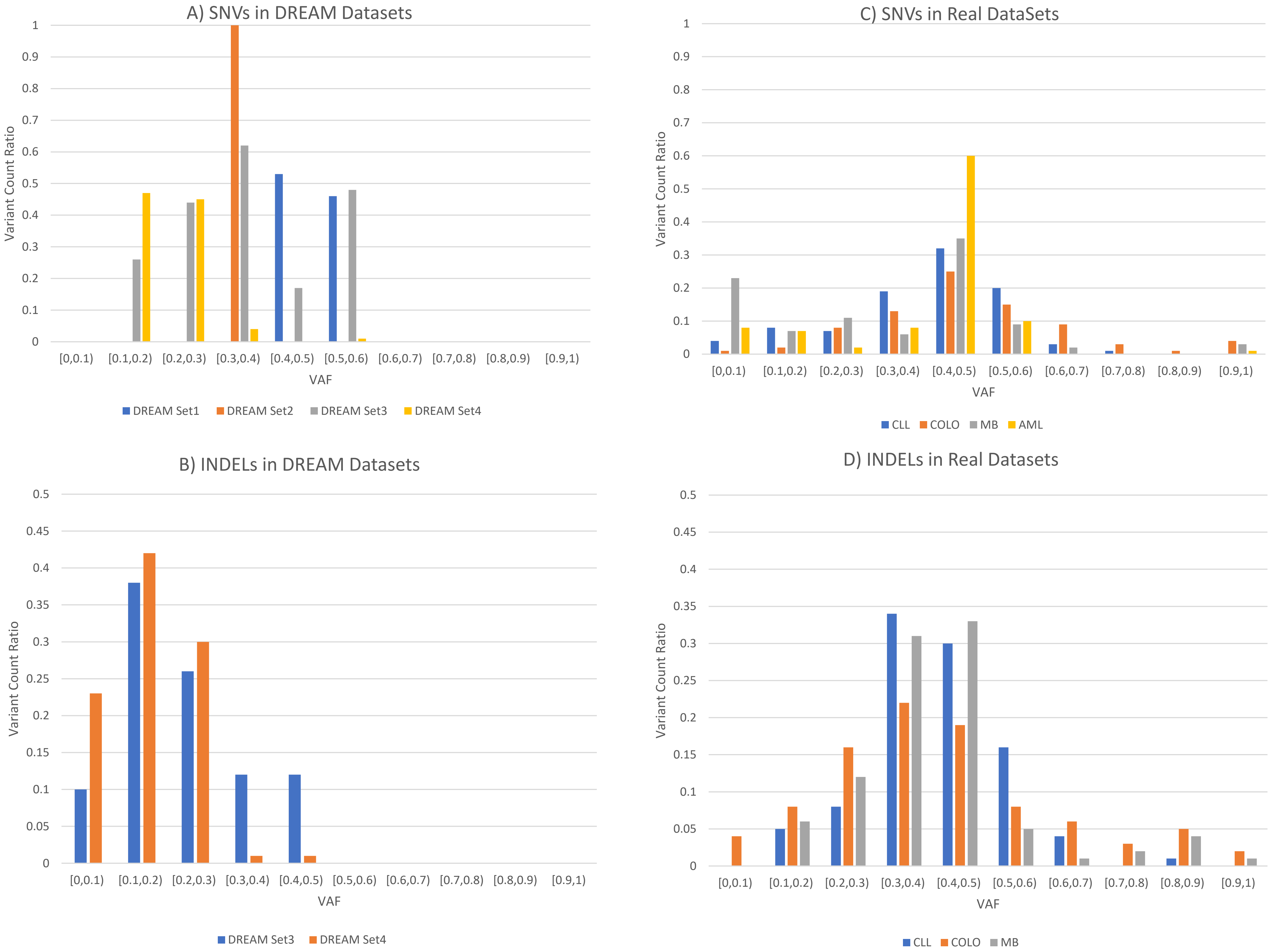

A) SNVs in DREAM Datasets
Variant Count Ratio
1
0.9
0.8
0.7
0.6
0.5
0.4
0.3
0.2
0.1
0
[0,0.1)
[0.1,0.2)
[0.2,0.3)
[0.3,0.4)
[0.4,0.5)
[0.5,0.6)
[0.6,0.7)
[0.7,0.8)
[0.8,0.9)
[0.9,1)
VAF
DREAM Set1
DREAM Set2
DREAM Set3
DREAM Set4
C) SNVs in Real DataSets
Variant Count Ratio
1
0.9
0.8
0.7
0.6
0.5
0.4
0.3
0.2
0.1
0
[0,0.1)
[0.1,0.2)
[0.2,0.3)
[0.3,0.4)
[0.4,0.5)
[0.5,0.6)
[0.6,0.7)
[0.7,0.8)
[0.8,0.9)
[0.9,1)
VAF
CLL
COLO
MB
AML
B) INDELs in DREAM Datasets
Variant Count Ratio
0.5
0.45
0.4
0.35
0.3
0.25
0.2
0.15
0.1
0.05
0
[0,0.1)
[0.1,0.2)
[0.2,0.3)
[0.3,0.4)
[0.4,0.5)
[0.5,0.6)
[0.6,0.7)
[0.7,0.8)
[0.8,0.9)
[0.9,1)
VAF
DREAM Set3
DREAM Set4
D) INDELs in Real Datasets
Variant Count Ratio
0.5
0.45
0.4
0.35
0.3
0.25
0.2
0.15
0.1
0.05
0
[0,0.1)
[0.1,0.2)
[0.2,0.3)
[0.3,0.4)
[0.4,0.5)
[0.5,0.6)
[0.6,0.7)
[0.7,0.8)
[0.8,0.9)
[0.9,1)
VAF
CLL
COLO
MB

Figure S2

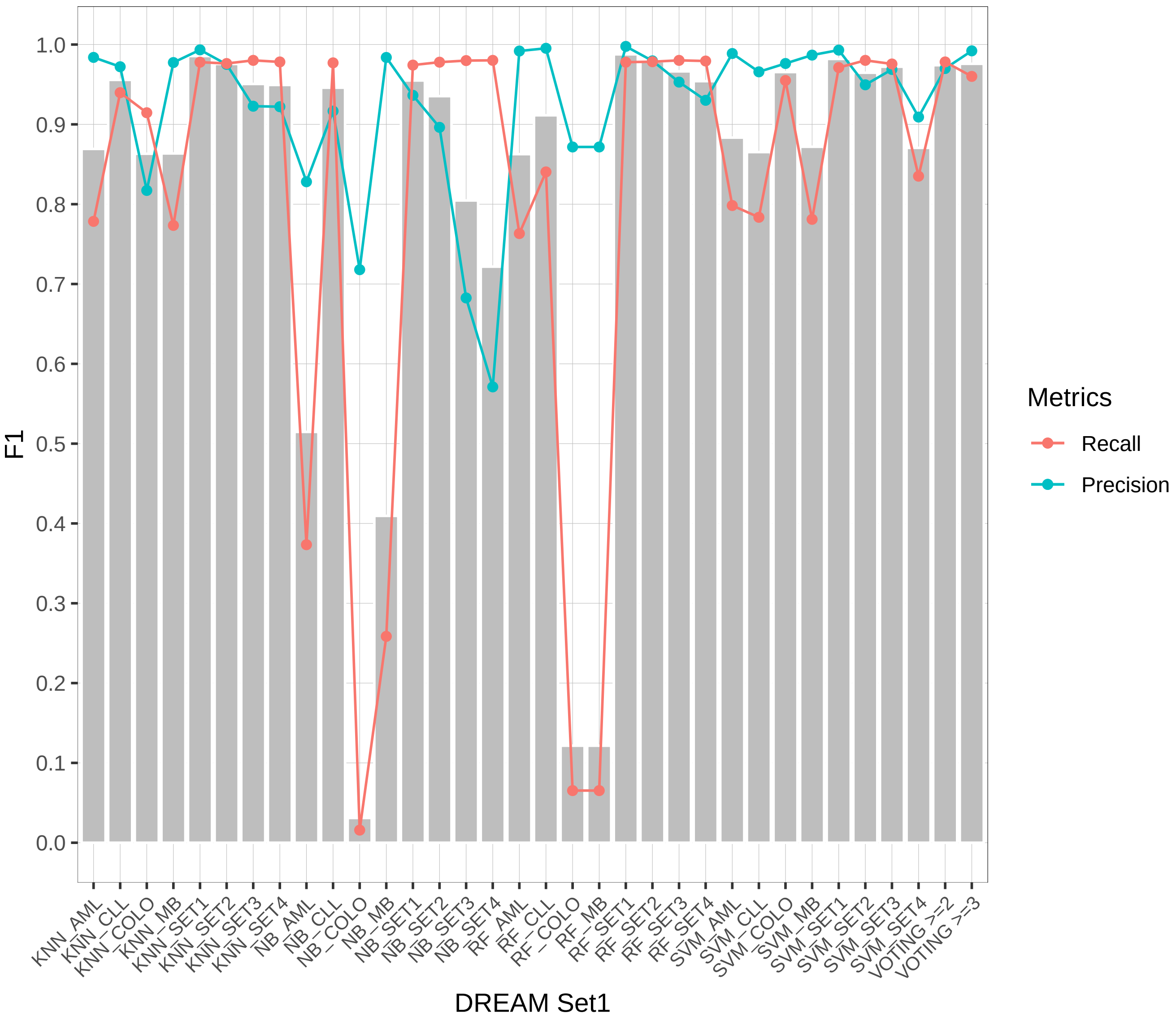

F1
1.0
0.9
0.8
0.7
0.6
0.5
0.4
0.3
0.2
0.1
0.0
KNN_AML
KNN_CLL
KNN_COLO
KNN_MB
KNN_SET1
KNN_SET2
KNN_SET3
KNN_SET4
NB_AML
NB_CLL
NB_COLO
NB_MB
NB_SET1
NB_SET2
NB_SET3
NB_SET4
RF_AML
RF_CLL
RF_COLO
RF_MB
RF_SET1
RF_SET2
RF_SET3
RF_SET4
SVM_AML
SVM_CLL
SVM_COLO
SVM_MB
SVM_SET1
SVM_SET2
SVM_SET3
SVM_SET4
VOTING >=2
VOTING >=3
DREAM Set1
Metrics
Recall
Precision

Figure S3

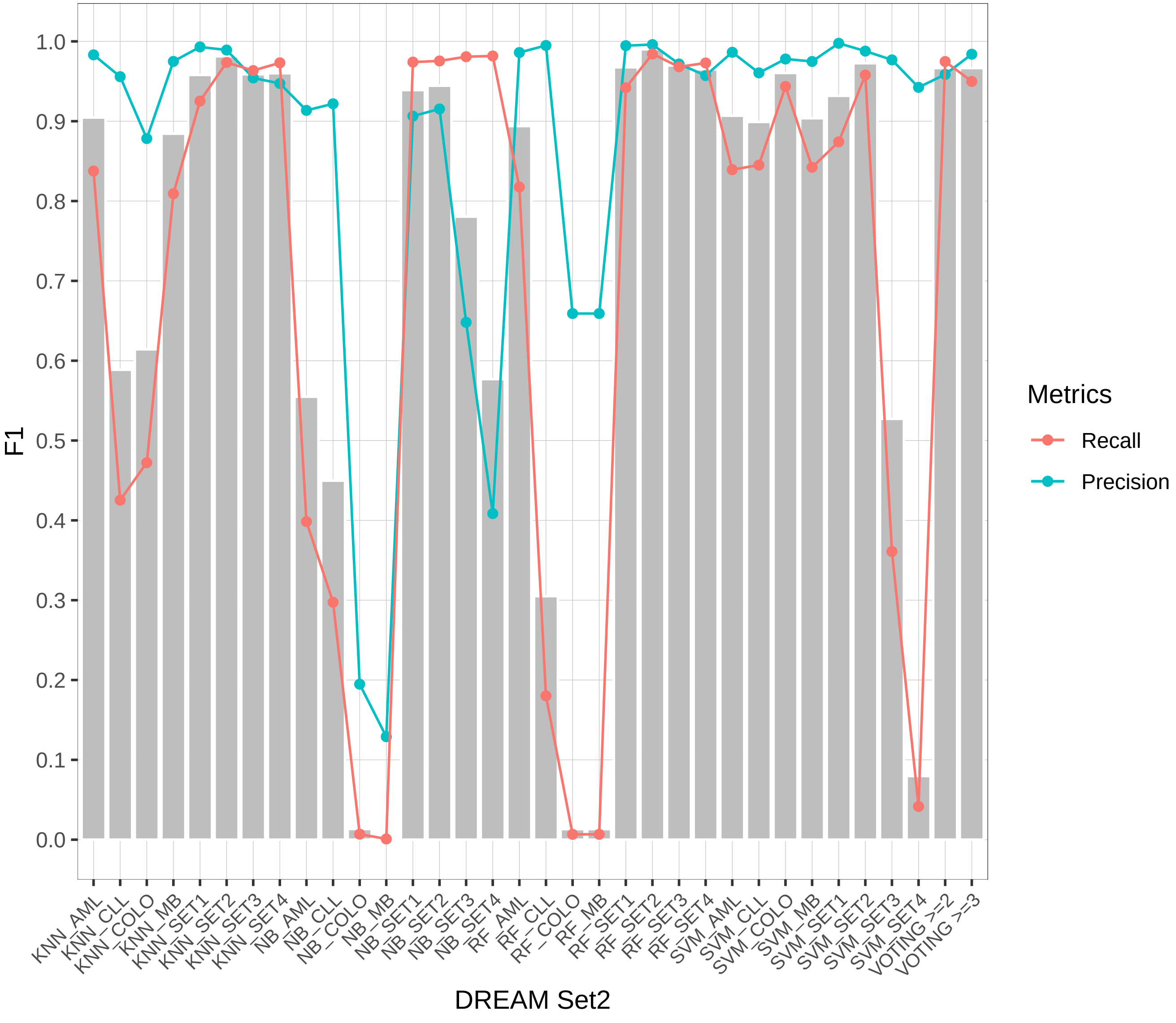

F1
1.0
0.9
0.8
0.7
0.6
0.5
0.4
0.3
0.2
0.1
0.0
KNN_AML
KNN_CLL
KNN_COLO
KNN_MB
KNN_SET1
KNN_SET2
KNN_SET3
KNN_SET4
NB_AML
NB_CLL
NB_COLO
NB_MB
NB_SET1
NB_SET2
NB_SET3
NB_SET4
RF_AML
RF_CLL
RF_COLO
RF_MB
RF_SET1
RF_SET2
RF_SET3
RF_SET4
SVM_AML
SVM_CLL
SVM_COLO
SVM_MB
SVM_SET1
SVM_SET2
SVM_SET3
SVM_SET4
VOTING >=2
VOTING >=3
DREAM Set2
Metrics
Recall
Precision

Figure S4

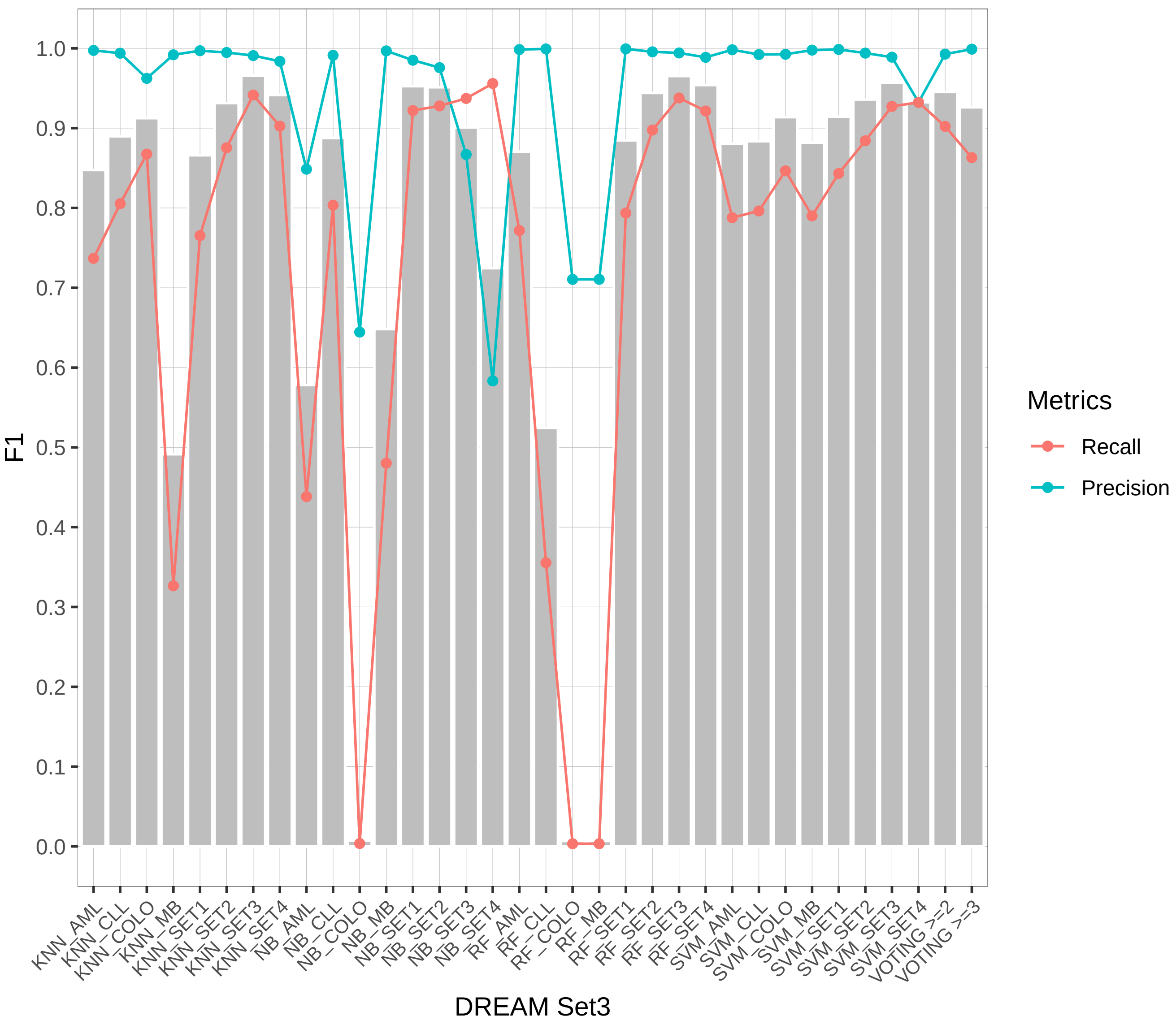

F1
1.0
0.9
0.8
0.7
0.6
0.5
0.4
0.3
0.2
0.1
0.0
KNN_AML
KNN_CLL
KNN_COLO
KNN_MB
KNN_SET1
KNN_SET2
KNN_SET3
KNN_SET4
NB_AML
NB_CLL
NB_COLO
NB_MB
NB_SET1
NB_SET2
NB_SET3
NB_SET4
RF_AML
RF_CLL
RF_COLO
RF_MB
RF_SET1
RF_SET2
RF_SET3
RF_SET4
SVM_AML
SVM_CLL
SVM_COLO
SVM_MB
SVM_SET1
SVM_SET2
SVM_SET3
SVM_SET4
VOTING >=2
VOTING >=3
DREAM Set3
Metrics
Recall
Precision

Figure S5

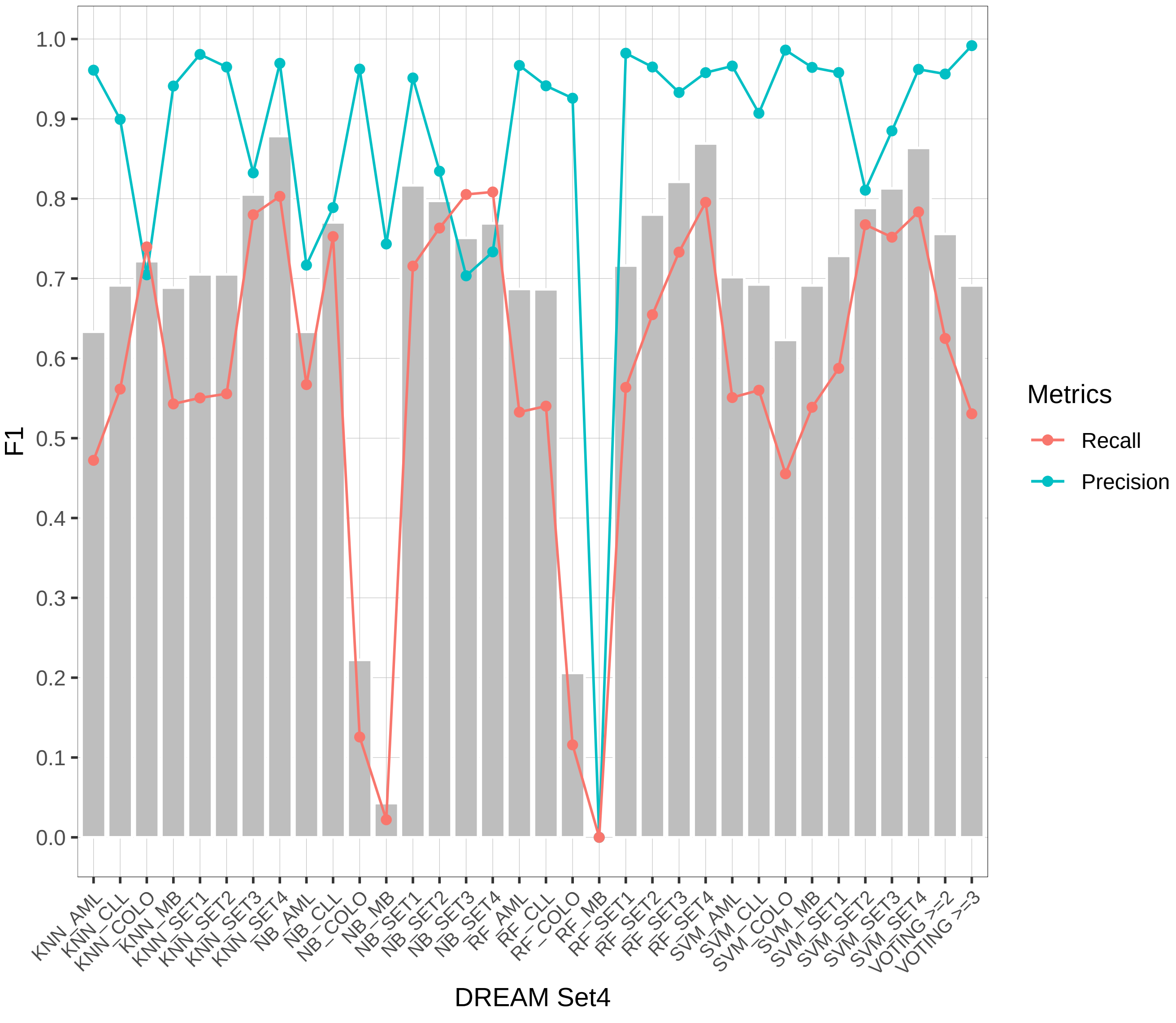
F1
DREAM Set4
Metrics
Recall
Precision
KNN_AML
KNN_CLL
KNN_COLO
KNN_MB
KNN_SET1
KNN_SET2
KNN_SET3
KNN_SET4
NB_AML
NB_CLL
NB_COLO
NB_MB
NB_SET1
NB_SET2
NB_SET3
NB_SET4
RF_AML
RF_CLL
RF_COLO
RF_MB
RF_SET1
RF_SET2
RF_SET3
RF_SET4
SVM_AML
SVM_CLL
SVM_COLO
SVM_MB
SVM_SET1
SVM_SET2
SVM_SET3
SVM_SET4
VOTING >=2
VOTING >=3

Figure S6

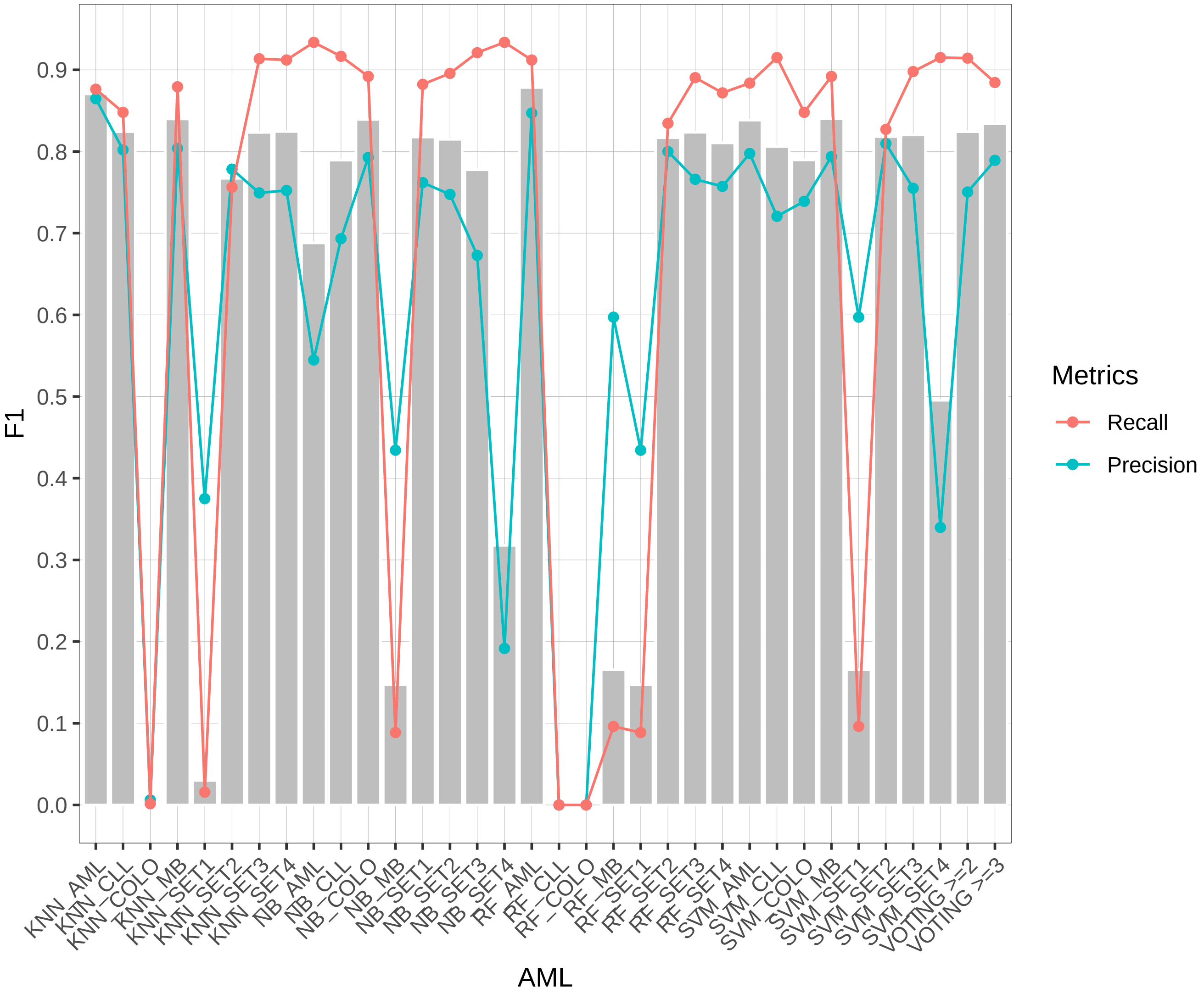

F1
0.0
0.1
0.2
0.3
0.4
0.5
0.6
0.7
0.8
0.9
KNN_AML
KNN_CLL
KNN_COLO
KNN_MB
KNN_SET1
KNN_SET2
KNN_SET3
KNN_SET4
NB_AML
NB_CLL
NB_COLO
NB_MB
NB_SET1
NB_SET2
NB_SET3
NB_SET4
RF_AML
RF_CLL
RF_COLO
RF_MB
RF_SET1
RF_SET2
RF_SET3
RF_SET4
SVM_AML
SVM_CLL
SVM_COLO
SVM_MB
SVM_SET1
SVM_SET2
SVM_SET3
SVM_SET4
VOTING >=2
VOTING >=3
AML
Metrics
Recall
Precision

Figure S7

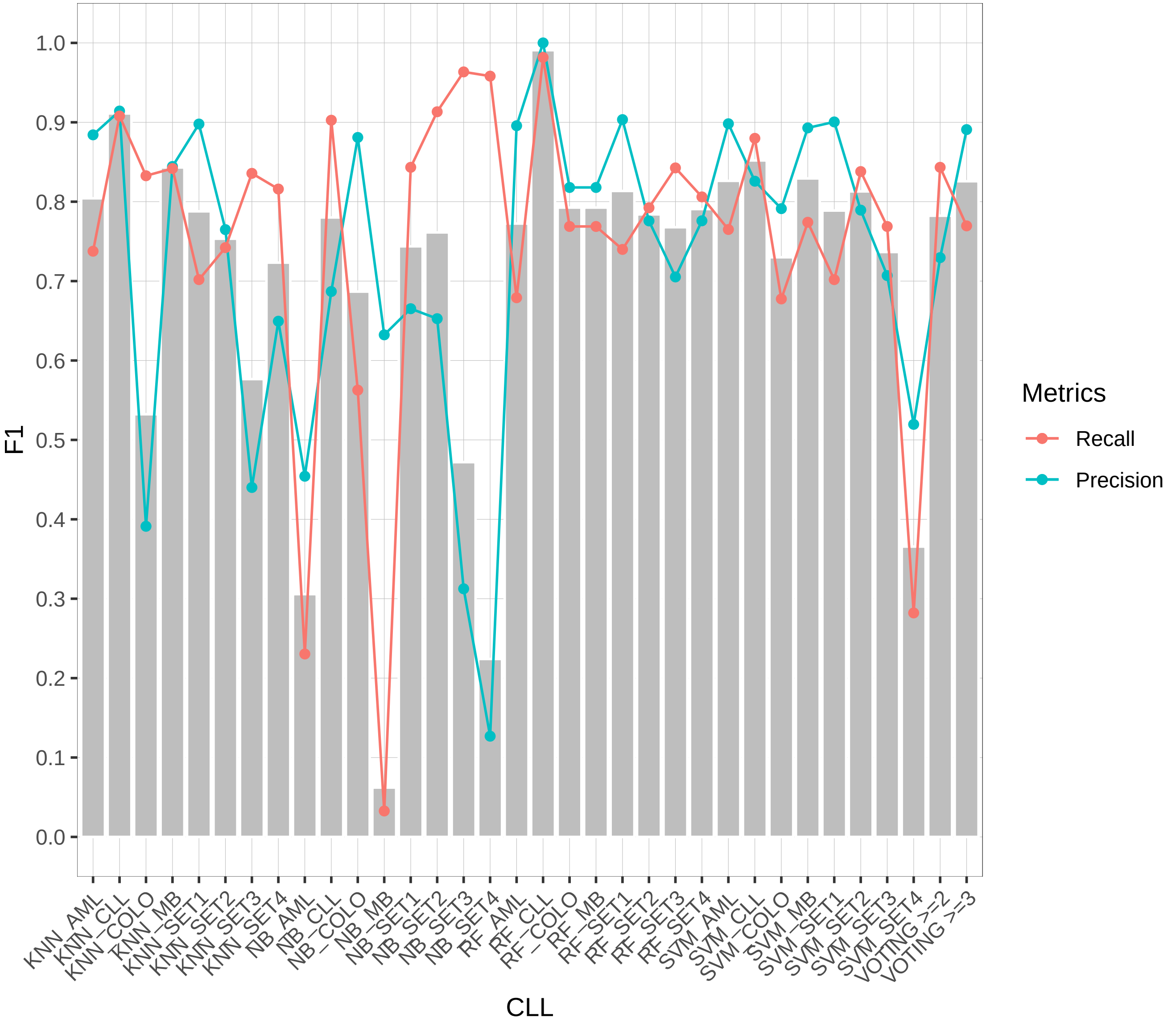
F1
1.0
0.9
0.8
0.7
0.6
0.5
0.4
0.3
0.2
0.1
0.0
KNN_AML
KNN_CLL
KNN_COLO
KNN_MB
KNN_SET1
KNN_SET2
KNN_SET3
KNN_SET4
NB_AML
NB_CLL
NB_COLO
NB_MB
NB_SET1
NB_SET2
NB_SET3
NB_SET4
RF_AML
RF_CLL
RF_COLO
RF_MB
RF_SET1
RF_SET2
RF_SET3
RF_SET4
SVM_AML
SVM_CLL
SVM_COLO
SVM_MB
SVM_SET1
SVM_SET2
SVM_SET3
SVM_SET4
VOTING >=2
VOTING >=3
CLL
Metrics
Recall
Precision

Figure S8

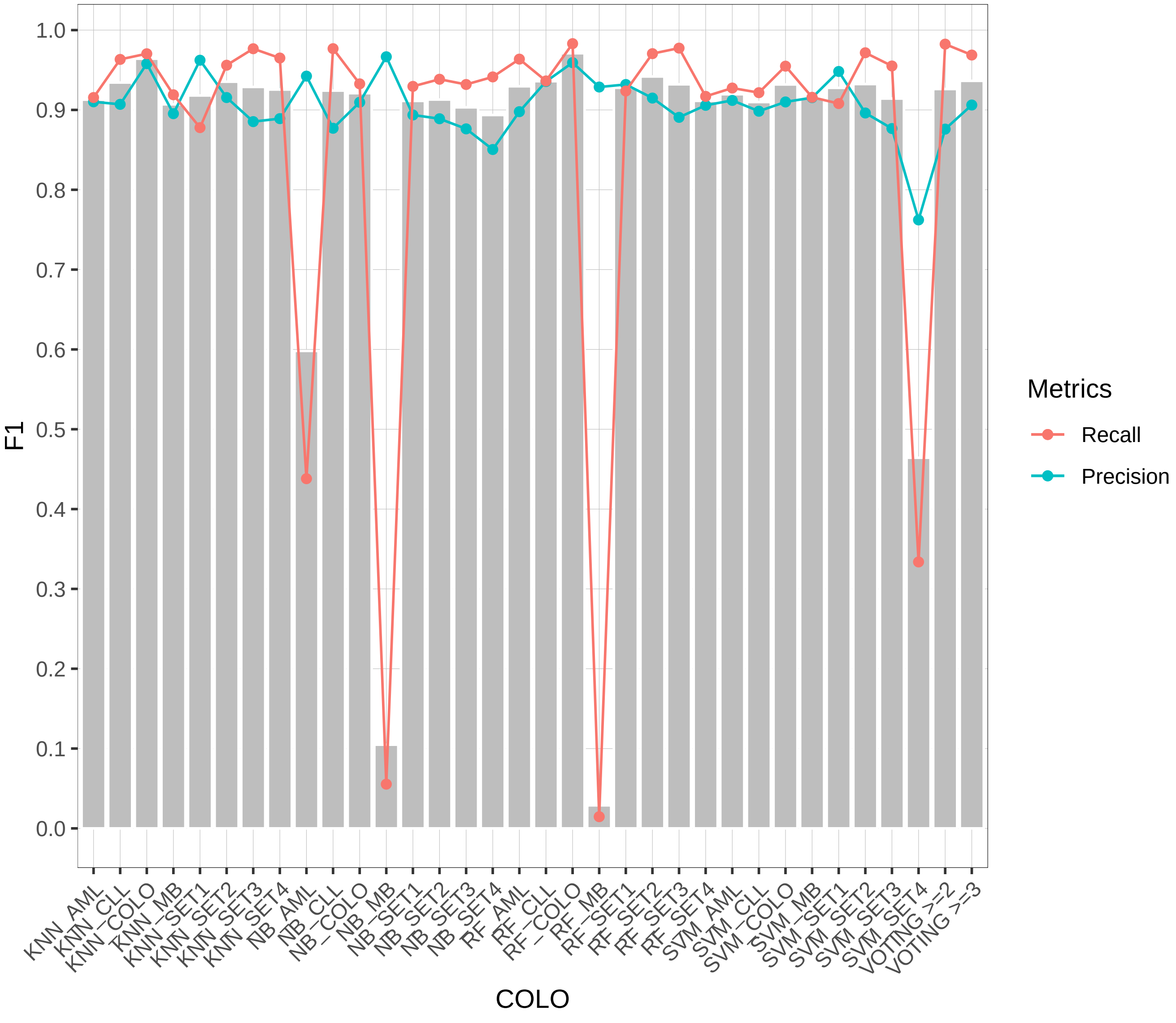

F1
COLO
Metrics
Recall
Precision
1.0
0.9
0.8
0.7
0.6
0.5
0.4
0.3
0.2
0.1
0.0
KNN_AML
KNN_CLL
KNN_COLO
KNN_MB
KNN_SET1
KNN_SET2
KNN_SET3
KNN_SET4
NB_AML
NB_CLL
NB_COLO
NB_MB
NB_SET1
NB_SET2
NB_SET3
NB_SET4
RF_AML
RF_CLL
RF_COLO
RF_MB
RF_SET1
RF_SET2
RF_SET3
RF_SET4
SVM_AML
SVM_CLL
SVM_COLO
SVM_MB
SVM_SET1
SVM_SET2
SVM_SET3
SVM_SET4
VOTING >=2
VOTING >=3

Figure S9

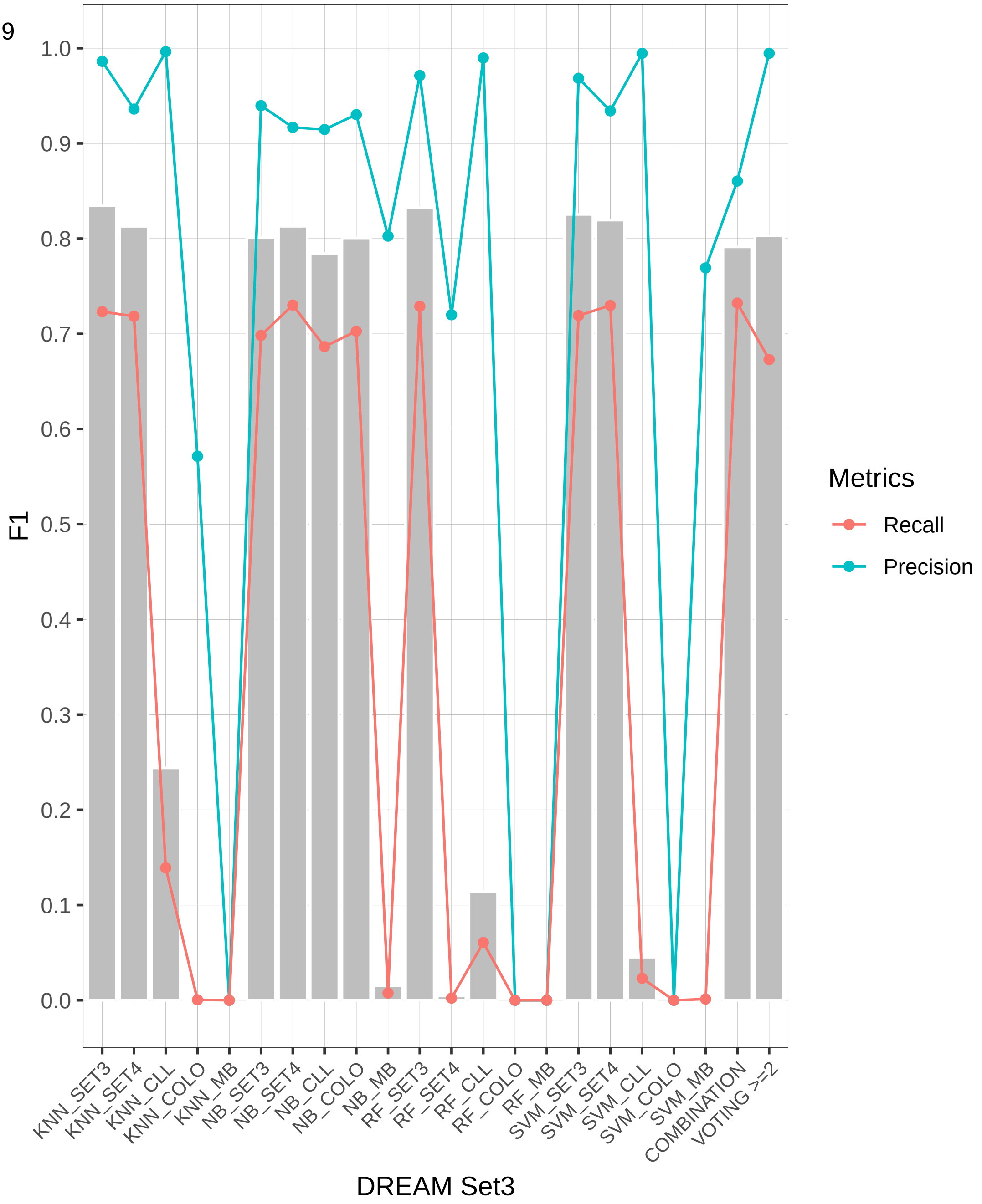
1.0
0.9
0.8
0.7
0.6
0.5
0.4
0.3
0.2
0.1
0.0
F1
KNN_SET3
KNN_SET4
KNN_CLL
KNN_COLO
KNN_MB
NB_SET3
NB_SET4
NB_CLL
NB_COLO
NB_MB
RF_SET3
RF_SET4
RF_CLL
RF_COLO
RF_MB
SVM_SET3
SVM_SET4
SVM_CLL
SVM_COLO
SVM_MB
COMBINATION
VOTING >=2
DREAM Set3
Metrics
Recall
Precision

Figure S10

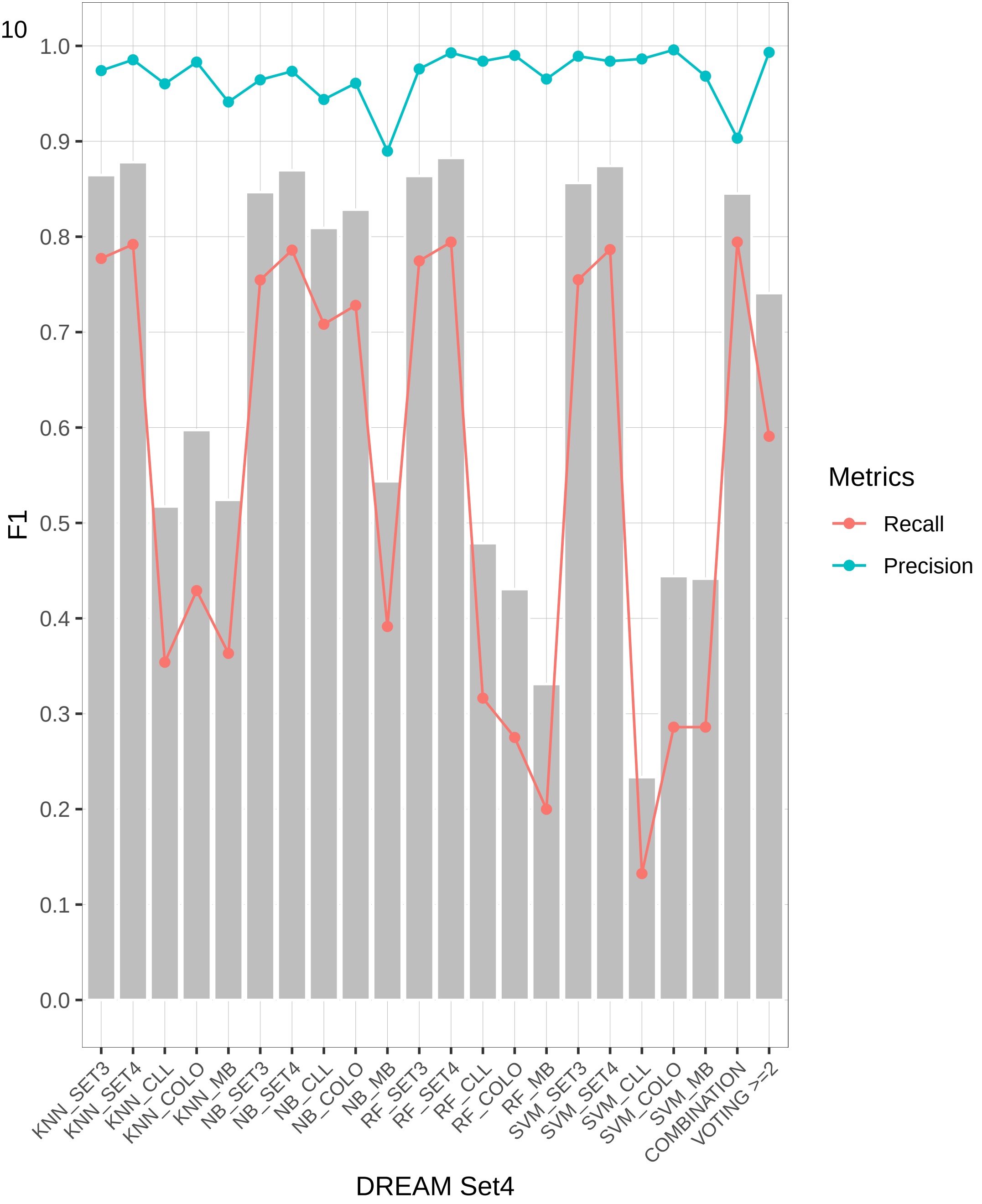

F1
1.0
0.9
0.8
0.7
0.6
0.5
0.4
0.3
0.2
0.1
0.0
KNN_SET3
KNN_SET4
KNN_CLL
KNN_COLO
KNN_MB
NB_SET3
NB_SET4
NB_CLL
NB_COLO
NB_MB
RF_SET3
RF_SET4
RF_CLL
RF_COLO
RF_MB
SVM_SET3
SVM_SET4
SVM_CLL
SVM_COLO
SVM_MB
COMBINATION
VOTING >=2
DREAM Set4
Metrics
Recall
Precision

Figure S11

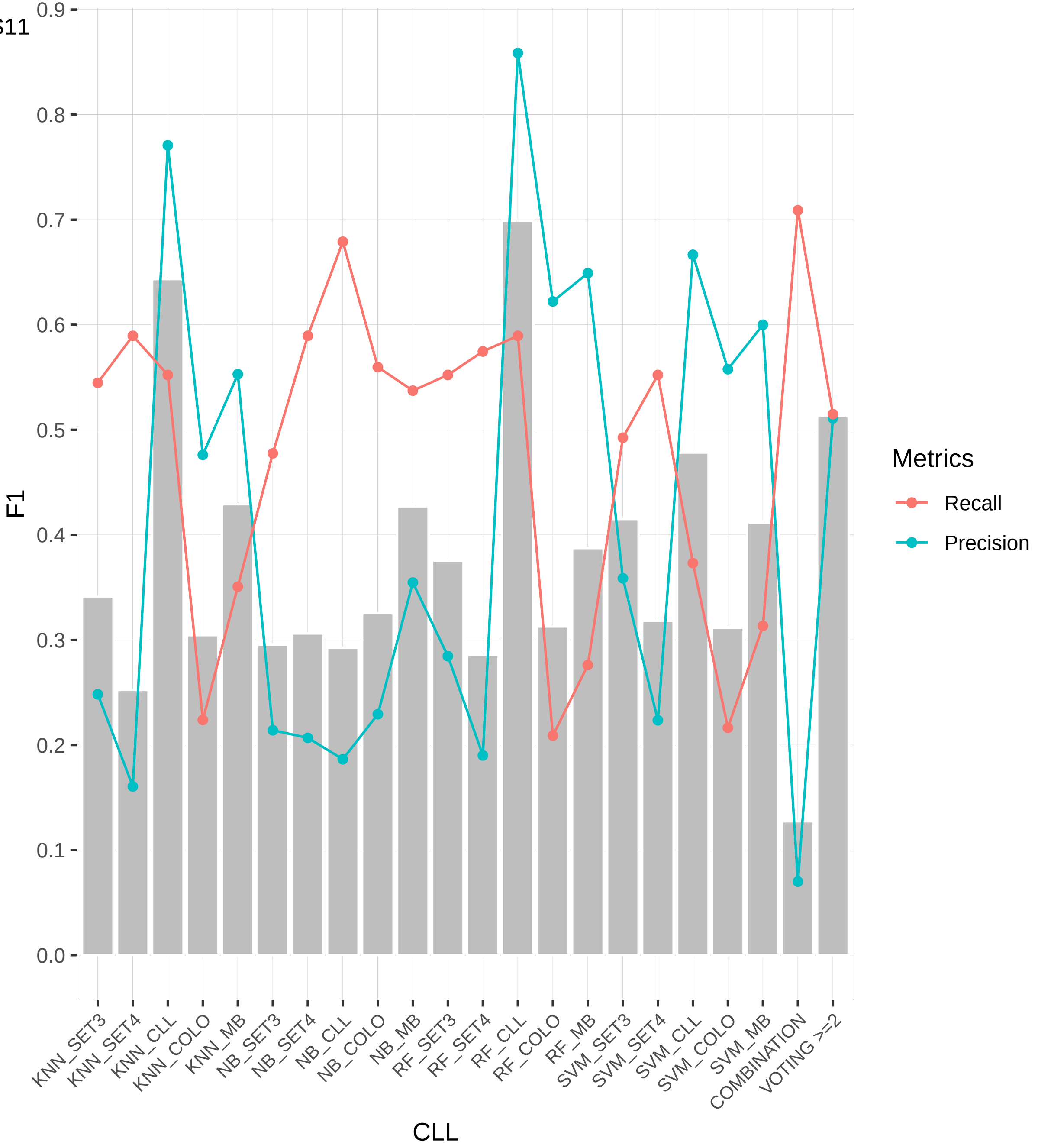

F1
0.0
0.1
0.2
0.3
0.4
0.5
0.6
0.7
0.8
0.9
KNN_SET3
KNN_SET4
KNN_CLL
KNN_COLO
KNN_MB
NB_SET3
NB_SET4
NB_CLL
NB_COLO
NB_MB
RF_SET3
RF_SET4
RF_CLL
RF_COLO
RF_MB
SVM_SET3
SVM_SET4
SVM_CLL
SVM_COLO
SVM_MB
COMBINATION
VOTING >=2
CLL
Metrics
Recall
Precision

Figure S12

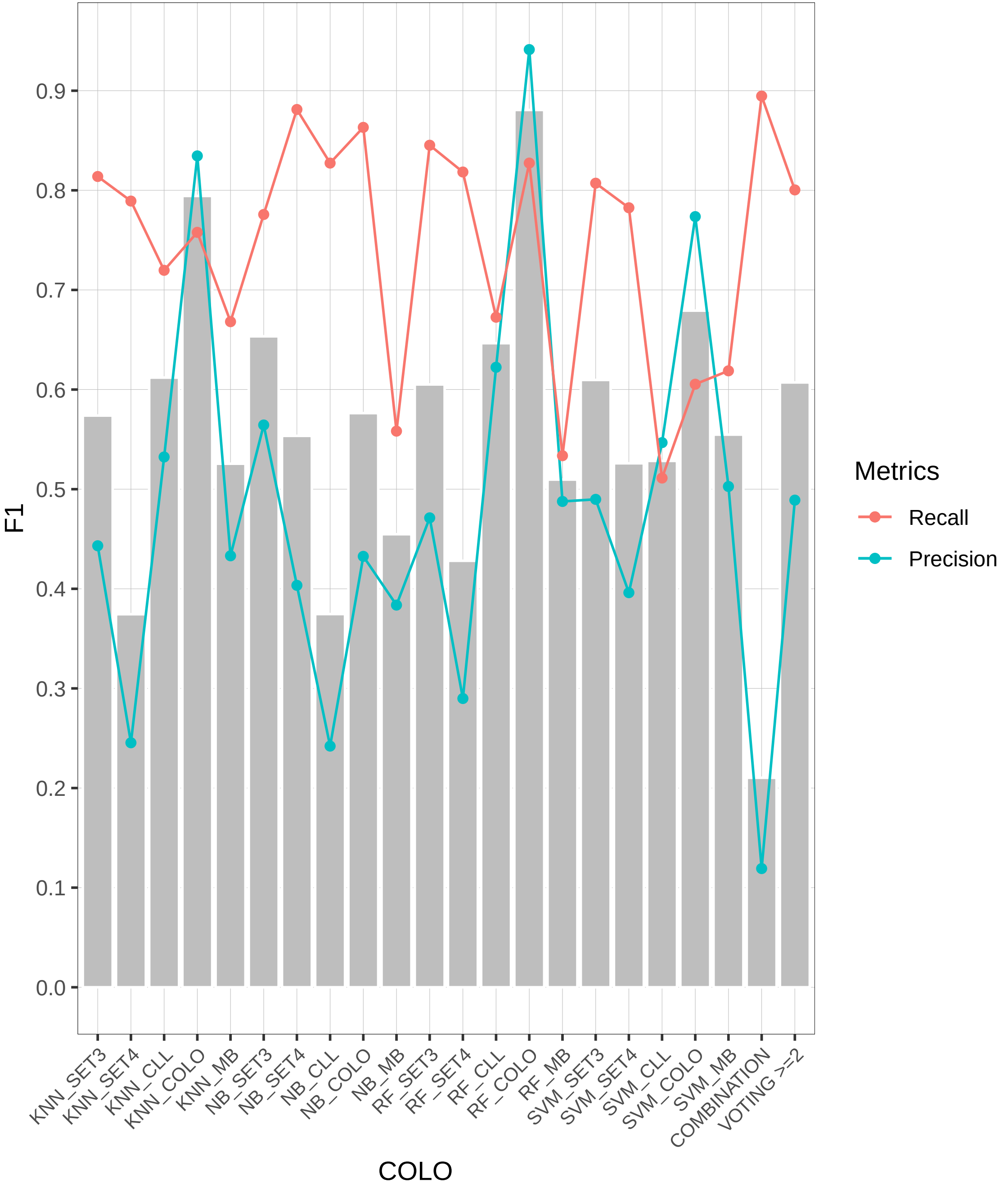

F1
0.0
0.1
0.2
0.3
0.4
0.5
0.6
0.7
0.8
0.9
KNN_SET3
KNN_SET4
KNN_CLL
KNN_COLO
KNN_MB
NB_SET3
NB_SET4
NB_CLL
NB_COLO
NB_MB
RF_SET3
RF_SET4
RF_CLL
RF_COLO
RF_MB
SVM_SET3
SVM_SET4
SVM_CLL
SVM_COLO
SVM_MB
COMBINATION
VOTING >=2
COLO
Metrics
Recall
Precision

Figure S13

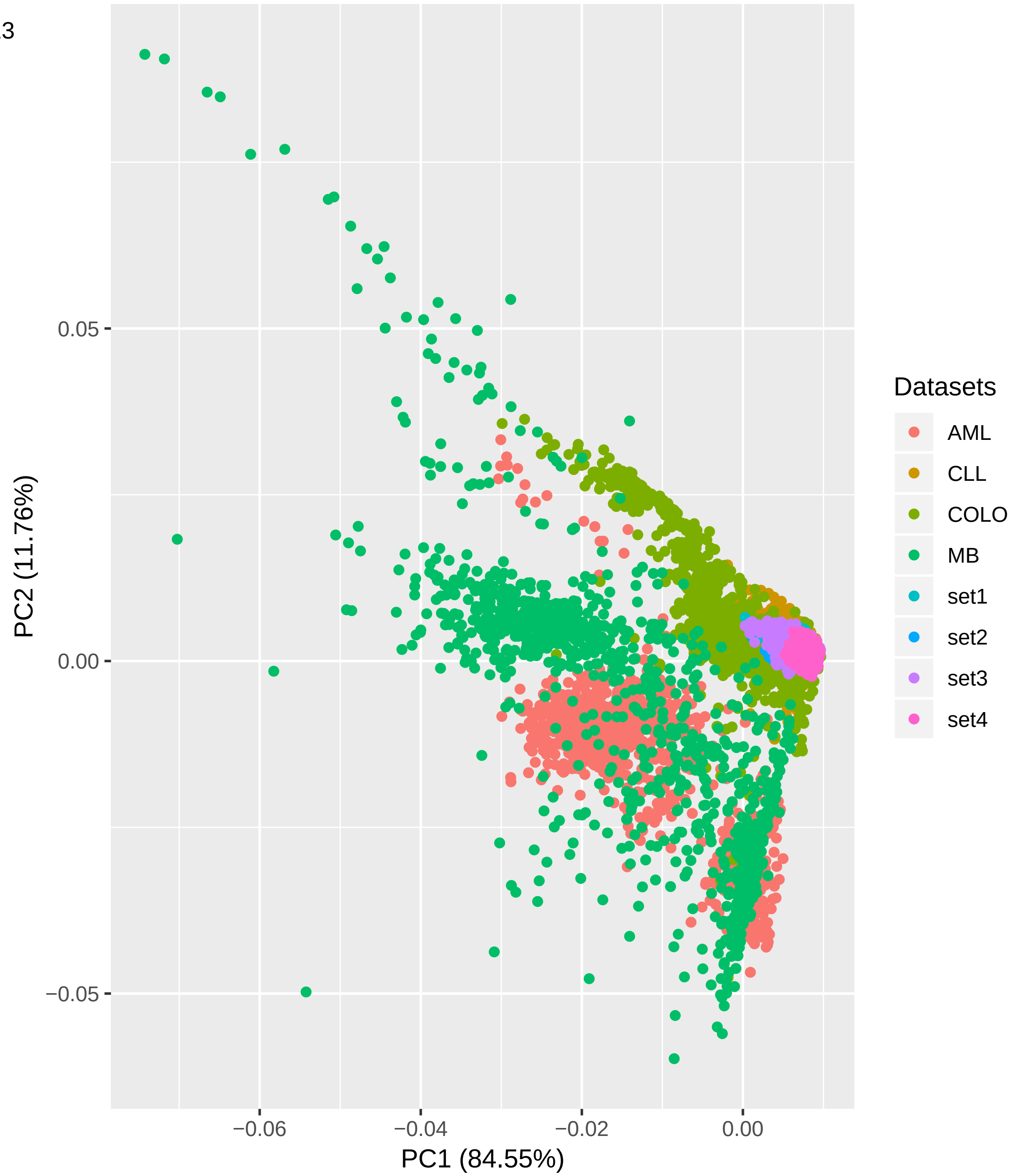
PC2 (11.76%)
0.05
0.00
−0.05
−0.06
−0.04
−0.02
0.00
PC1 (84.55%)
Datasets
AML
CLL
COLO
MB
set1
set2
set3
set4
