# Supplementary material for: SomaticCombiner: improving the performance of somatic variant calling based on evaluation tests and a consensus approach
Source: Sci Rep. 2020 Jul 30;10:12898. doi: 10.1038/s41598-020-69772-8 (PMC7393490; doi:10.1038/s41598-020-69772-8)
Supplement: Supplementary file 2 — Supplementary information. [file 41598_2020_69772_MOESM2_ESM.docx]

**SomaticCombiner: improving the performance of somatic variant calling based on evaluation tests and a consensus approach**

**Supplementary Information**

Mingyi Wang^1, *^, Wen Luo^1^, Kristine Jones^1^, Xiaopeng Bian^2^, Russell Williams^1^, Herbert Higson^1^, Dongjing Wu^1^, Belynda Hicks^1^, Meredith Yeager^1^, Bin Zhu^1, *^

^1^ Cancer Genomics Research Laboratory, Division of Cancer Epidemiology and Genetics, Frederick National Laboratory for Cancer Research, Frederick, MD, 20877, USA;

^2^Center for Biomedical Informatics and Information Technology, National Cancer Institute, Rockville, MD, 20850, USA

**Results**

**Evaluation of individual callers using WGS datasets**

For SNV calling in the DREAM dataset 1, 2, and 3 (Fig. 1A and Table S1), all callers achieved high recall values (>0.709), but precision values varied. In these three datasets, LoFreq, MuSE, Mutect2 and Strelka achieved higher precision values and better *F*1 scores compared to the other four callers (Fig. 1 and Table S1). The prediction pattern in the DREAM set 4 is different compared to the other three DREAM datasets (Fig. 1). In this dataset, MuTect2 showed the highest *F*1-score (0.825), followed by MuTect (0.782) and VarDict (0.761), and these three callers showed higher sensitivity than other callers.

For SNV calling in the real cancer datasets (Fig. 2 and Table S1), most callers yielded high *F*1 scores (>0.871) in the COLO dataset, but SomaticSniper performed poorly. For the remaining three real datasets, *F*1 scores of all callers were lower than the synthetic DREAM sets 1-3. From Fig. S1, we can see that all four real datasets do include true SNVs with low VAFs (<0.2) and high VAFs (>0.6) that are not presented in the DREAM datasets. Although the real datasets are more difficult for all callers, LoFreq, MuSE, and Strelka still performed better than other ones in terms of *F*1 score (Fig. 2B).

In general, LoFreq, MuSE and Strelka returned fewer SNVs and yielded higher precision values compared to other callers, which indicated that these three callers are more conservative and have higher specificity. MuTect and MuTect2 showed moderate *F*1 scores except for the DREAM set4. VarDict, VarScan and SomaticSniper have lower precision values than other callers, which negatively impact their overall *F*1 scores.

We also ran INDEL calling tests on five datasets (DREAM set3, set4, CLL, COLO and MB), as they were the only ones provided with the high confidence INDEL call sets. From the results (Fig. 3 and Table S2), we see INDEL calling is much more challenging than SNV calling, especially in the real datasets. Correct INDEL calling is highly dependent on accurate alignment, and MuTect2 is the only caller that applies local de novo assembly around indels to optimize indel calling. The *F*1 scores only ranged from 0.039 to 0.553 in the real datasets (Fig. 3B and Table S2). In the real datasets, the reduced performance is mainly caused by very low precision values ranging between 0.020-0.459 (Fig. 3A and Table S2) for all callers. The low precisions may also be attributed to the incompleteness of true INDELs in the three real datasets. Among the five INDEL callers MuTect2 consistently showed the best performance in terms of *F*1 score compared to all others except it achieved second-best in the MB dataset. Overall, Strelka and LoFreq performed as second or third best callers in DREAM set3, CLL and COLO. VarDict ranked second, just below MuTect2 in the DREAM set4, and VarScan was best in MB. VarDict and VarScan have lower precision values than the other three callers in four datasets, except for MB.

**Evaluation of individual callers using deep targeted sequencing datasets**

We then moved to the assessment of the deep targeted sequencing datasets, which include spiked-in NA12878 with six purity levels (1%, 2%, 5%, 10%, 20% and 50%) and four combinations (two “tumor” NA12878 spiked-in replicates compared against two “normal” NA24385 replicates) at each level. For each purity level, the average evaluation metrics from four combinations were calculated and shown in Fig. 4. Table S3 shows the performance of all pairs. Note that some callers failed to return any variants for some pairs, and thus, the results of those pairs are not listed. The deep read depth (~1600×) is supposed to yield higher sensitivity for variant detection at low VAFs. This dataset, therefore, provides us an opportunity to evaluate the effect of read depth, tumor purity and VAFs on each caller.

In the SNV calling (Fig. 4A), for higher percentage purity levels (50% and 20% of NA12878), five callers showed a similar improved performance in average *F*1 scores (four combinations): Strelka (0.980 and 0.984), LoFreq (0.993 and 0.963), VarDict (0.961 and 0.966), MuTect (0.956 and 0.951), and MuSE (0.917 and 0.919). SomaticSniper yielded the highest *F*1 score (0.995) among all callers for the 50% NA12878 level but dropped significantly to 0.339 for 20% NA12878. For 10% NA12878, Strelka still maintained an *F*1 score of 0.984, followed by VarDict (0.964), MuSE (0.920), and MuTect (0.916), while LoFreq only yielded the *F*1 score 0.729 at this level. For 5% NA12878, the performance varied; VarDict and Strelka maintained the *F*1 scores 0.963 and 0.967, while Muse, MuTect, and LoFreq dropped to 0.771, 0.346 and 0.182 due to decreased sensitivities. Strelka and VarDict performed similarly, achieving 0.335 and 0.378 for 2% NA12878, while LoFreq, MuTect, and MuSE failed to detect most of the true SNVs. Compared to other callers, MuTect2 showed a different tendency; it demonstrated high sensitivity in extremely low purity samples, with an *F*1 score of 0.607 in 2% and 0.396 in 1%, respectively. However, the sensitivity of MuTect2 remained steady as tumor purity levels increased from 5% to 50%. VarScan was another outlier in the deep targeted sequencing data, as it only called SNVs in 50% NA12878 and failed to call any SNVs in other samples, suggesting VarScan has low sensitivity for low VAFs.

From the analysis, Strelka and VarDict showed a better overall performance in the deep targeted sequencing tests, and the performance of these two callers was very similar as purity levels of NA12878 decrease.

For INDEL calling (Fig. 4B and Table S4), out of five INDEL callers only three (VarDict, MuTect2, and LoFreq) worked well for all levels in this dataset. LoFreq performed better than the other two at 1%, 2%, 5%, and 10%, while VarDict outperformed the others at 10% and 25%. As we saw in SNV calling, VarScan could only detect INDELs at 50% and failed to call any INDELs when the purity levels were lower than 50%. Strelka did not return any “PASS” INDELs in the NA12878 high confidence regions.

To evaluate the impact of sequence coverage on different callers, for each purity levels, we also downsampled a NA12878 spiked-in replicate to four coverages levels (100×, 200×, 500× and 1000×) and a NA24385 replicate to two coverage levels (50× and 100×), then ran somatic calling for these downsampled pairs. From the SNV calling results for each tumor purity level (Table S5), the overall performance improved as the tumor read depth increased. However, at the 50% tumor purity level, the impact of tumor depth was minimal for all callers. For the INDEL calling (Table S6), it is interesting that *F*1 scores did not increase as the tumor read depth increased for 10%, 20% and 50% NA12878. In general, at these three higher purity levels, the samples with higher depth returned more INDELs but did not improve in overall performance. In contrast, we observed increasing *F*1 scores with higher tumor coverage at lower purity levels 1%, 2% and 5% NA12878 for most callers except that two outliers (VarScan and Strelka) have very low sensitivities. For INDEL calling, we also observed that the normal samples with a higher coverage level (100×) returned fewer variants but slightly increased *F*1 scores when compared to lower coverage (50×) normal samples. However, for SNV calling, the impact of the coverage in normal samples was minimal.

**Evaluation of individual callers using WES datasets**

As an extension of the evaluation of deep targeted sequencing data, we used the NA12878-NA11840 dilution WES series datasets ^1^ to evaluate the callers further using data with lower coverage but targeting all coding regions. Similar in concept to the deep targeted sequencing data, the dataset included 11 pairs, which comprised of the NA12878 dilution series (11 purity levels from 0.2% to 100%) and NA11840 to mimic tumor-normal sample pairs.

From the SNV calling tests (Fig. 5A and Table S7), we can see most callers (LoFreq, MuSE, MuTect, VarScan, SomaticSniper, and Strelka) showed very similar *F*1 scores, ranging from 0.725 to 0.823 in the 60%, 80%, and 100% NA12878 purity levels. The *F*1 scores of VarScan and SomaticSniper dropped more quickly than the other four callers in 40% and 20% NA12878. This is in accord with their low sensitivities in the deep targeted sequencing data when tumor fractions decreased to 20%. The performances of all callers degraded as the purity level went below 10%. MuTect2, VarDict and Strelka still showed their strength in detecting low VAF SNVs when NA12878 is between 1% - 5%.

For SNV calling, Strelka stood out, showing the best overall performance in all samples, even achieving similar performance to MuTect2 in low VAF sites. MuTect2 showed slightly lower *F*1 scores than other callers at higher purity levels due to lower sensitivity, which is also consistent with observations from the deep targeted sequencing data. VarDict showed a different calling pattern than the other callers in this dataset with consistently low precisions at all purity levels, which indicates that VarDict is very sensitive and returned a high number of calls, including many false positives.

For INDEL calling (Fig. 5B and Table S8), VarScan achieved the best performance in 60% - 100% NA12878. However, the recall values of VarScan dropped quickly in 20% and below, similar to its performance in the deep targeted sequencing data. These results confirmed that VarScan is impacted remarkedly by tumor purity levels. At lower purity levels (2% -40%), LoFreq showed higher recall values, but lower precisions than MuTect2, and these callers showed comparable *F*1 scores and achieved the overall best performance. As in SNV calling, VarDict showed high sensitivity at the cost of high false positives. All callers returned very few INDELs at ultra-low purity levels 0.2%-1%. Unlike its superior performance in SNV calling, Strelka showed very low sensitivities in INDEL calling for all samples; this is also consistent with its performance in the deep targeted sequencing data.

**Evaluation of our consensus approach using downsampled deep targeted sequencing data**

From the downsampled deep targeted sequencing data (Table S5 and S6), we compared our consensus approach with individual callers to further check its performance across various purity levels and coverage depths. The test results for all downsampled pairs are presented in Table S5 and S6, and the highest *F*1 scores for each pair are highlighted by bold font. From the results, the consensus ensemble approach achieved better overall performance than any individual caller across all coverage and tumor purity levels.

**References**

1 Callari, M. *et al.* Intersect-then-combine approach: improving the performance of somatic variant calling in whole exome sequencing data using multiple aligners and callers. *Genome medicine* **9**, 35, doi:10.1186/s13073-017-0425-1 (2017).
